# Impact of seasonal RTS,S/AS01_E_ vaccination plus seasonal malaria chemoprevention on the nutritional status of children in Burkina Faso and Mali

**DOI:** 10.1186/s12936-022-04077-x

**Published:** 2022-02-22

**Authors:** Jane Grant, Issaka Sagara, Issaka Zongo, Matthew Cairns, Rakiswendé Serge Yerbanga, Modibo Diarra, Charles Zoungrana, Djibrilla Issiaka, Frédéric Nikièma, Frédéric Sompougdou, Amadou Tapily, Mahamadou Kaya, Alassane Haro, Koualy Sanogo, Abdoul Aziz Sienou, Seydou Traore, Ismaila Thera, Hama Yalcouye, Irene Kuepfer, Paul Snell, Paul Milligan, Christian Ockenhouse, Opokua Ofori-Anyinam, Halidou Tinto, Abdoulaye Djimde, Daniel Chandramohan, Brian Greenwood, Alassane Dicko, Jean-Bosco Ouédraogo

**Affiliations:** 1 London School of Hygiene and Tropical Medicine, Keppel St., London, WC1E 7HT UK; 2 Malaria Research and Training Center, University of Science, Techniques, and Technologies of Bamako, Bamako, Mali; 3 Institut de Recherche en Sciences de La Santé, Bobo-Dioulasso, Burkina Faso; 4 PATH, Seattle, USA; 5 GlaxoSmithKline Vaccines, Rixensart, Belgium

**Keywords:** Malaria, Nutrition, Seasonal malaria chemoprevention, RTS,S/AS01_E_, Mali, Burkina Faso

## Abstract

**Background:**

A recent trial in Burkina Faso and Mali showed that combining seasonal RTS,S/AS01_E_ malaria vaccination with seasonal malaria chemoprevention (SMC) substantially reduced the incidence of uncomplicated and severe malaria in young children compared to either intervention alone. Given the possible negative effect of malaria on nutrition, the study investigated whether these children also experienced lower prevalence of acute and chronic malnutrition.

**Methods:**

In Burkina Faso and Mali 5920 children were randomized to receive either SMC alone, RTS,S/AS01_E_ alone, or SMC combined with RTS,S/AS01_E_ for three malaria transmission seasons (2017–2019). After each transmission season, anthropometric measurements were collected from all study children at a cross-sectional survey and used to derive nutritional status indicators, including the binary variables wasted and stunted (weight-for-height and height-for-age z-scores below − 2, respectively). Binary and continuous outcomes between treatment groups were compared by Poisson and linear regression.

**Results:**

In 2017, compared to SMC alone, the combined intervention reduced the prevalence of wasting by approximately 12% [prevalence ratio (PR) = 0.88 (95% CI 0.75, 1.03)], and approximately 21% in 2018 [PR = 0.79 (95% CI 0.62, 1.01)]. Point estimates were similar for comparisons with RTS,S/AS01_E_, but there was stronger evidence of a difference. There was at least a 30% reduction in the point estimates for the prevalence of severe wasting in the combined group compared to the other two groups in 2017 and 2018. There was no difference in the prevalence of moderate or severe wasting between the groups in 2019. The prevalence of stunting, low-MUAC-for-age or being underweight did not differ between groups for any of the three years. The prevalence of severe stunting was higher in the combined group compared to both other groups in 2018, and compared to RTS,S/AS01_E_ alone in 2017; this observation does not have an obvious explanation and may be a chance finding. Overall, malnutrition was very common in this cohort, but declined over the study as the children became older.

**Conclusions:**

Despite a high burden of malnutrition and malaria in the study populations, and a major reduction in the incidence of malaria in children receiving both interventions, this had only a modest impact on nutritional status. Therefore, other interventions are needed to reduce the high burden of malnutrition in these areas.

*Trial registration*: https://www.clinicaltrials.gov/ct2/show/NCT03143218, registered 8th May 2017.

**Supplementary Information:**

The online version contains supplementary material available at 10.1186/s12936-022-04077-x.

## Background

The malaria burden remains high in sub-Saharan Africa, with an estimated 228 million cases and 602,000 deaths in 2020 [1]. Six of the 10 countries prioritized by the World Health Organization (WHO) ‘High Burden–High Impact’ initiative are in the Sahelian or sub-Sahelian regions of Africa, where malaria transmission is very seasonal and still very high [2]. Seasonal Malaria Chemoprevention (SMC), the monthly administration of sulfadoxine-pyrimethamine plus amodiaquine to children under five years of age during the malaria transmission season, is an effective way of preventing malaria in young children in areas with seasonal malaria. SMC is estimated to reduce the incidence of clinical malaria in the 28 days following administration by around 70% when effectively deployed [3]. SMC is now being deployed widely in countries of the African Sahel and sub-Sahel. Nevertheless, malaria remains the most frequent cause of death and hospital admissions in young children in many countries where SMC is being implemented, despite high coverage with SMC and insecticide-treated bed nets and good access to effective diagnosis and treatment. Improved use of existing control tools and novel approaches are urgently needed if malaria is to be brought fully under control in these countries.

The RTS,S/AS01_E_ malaria vaccine is one such possibility. Taking advantage of the vaccine’s initially high but rapidly waning protective efficacy, it has been suggested that RTS,S/AS01_E_ could be used as a seasonal vaccine, with children who have been primed early in life given an annual booster injection shortly before each malaria transmission season, either in addition to or as a replacement for SMC [4]. This concept has recently been tested in a trial undertaken in 5920 children in Burkina Faso and Mali during the period 2017–2019. The trial found that the combination of RTS,S/AS01_E_ given together with SMC was markedly superior to either intervention given alone, with the combination of the two interventions reducing clinical episodes of malaria by 62.8%, severe malaria requiring hospitalization by 70.5%, and deaths from malaria by 72.9%, compared to the protection provided by SMC alone [5]. Additionally, RTS,S/AS01E alone was found to be non-inferior to SMC given alone.

In addition to malaria, countries of the African Sahel and sub-Sahel have a high burden of under-nutrition among children under five years of age [6]. The peak of the malaria transmission season coincides with the ‘hunger season’, the period before the harvest when the risk of acute malnutrition in children is highest. Despite this seasonal overlap, the relationship between malaria and malnutrition at the individual level is complicated. It is postulated that malaria infection may have an adverse effect on nutritional status due to appetite loss, vomiting and abnormal nutrient metabolism, with repeated clinical episodes contributing to chronic malnutrition [7, 8]. Analysing the interaction between malaria and malnutrition is also made difficult by the multifactorial causes of malnutrition and a potential bidirectional relationship between malaria and malnutrition.

A systematic review of observational studies found no consistent association between malaria and malnutrition [9]. However, within the review, eight studies showed some evidence of an association between either malaria infection or clinical malaria and malnutrition. For example, two studies in Kenya and Uganda reported an increased risk of stunting and being underweight in children and infants who had been infected with malaria [10, 11]. Furthermore, a study in The Gambia found a negative relationship between clinical malaria and weight gain, but not for gain in height [12]. Additionally, a recent large cohort study in Ethiopia found that malaria infection was a risk factor for stunting and wasting [13]. Comparisons across these studies is difficult due to substantial heterogeneity in study population, sample size, nutritional outcomes measured, follow-up time, as well as location, malaria epidemiology and a range of other confounding factors, and whether these factors were adjusted for in the analysis.

Malaria prevention had a positive impact on nutritional status in children under five years of age in a number of randomized control trials. Multiple trials of insecticide-treated bed nets in Africa have shown improvements in different measures of nutrition amongst children who received the trial intervention compared to the control groups [7, 14–16]. Furthermore, four trials of intermittent preventive treatment of malaria in children and infants in West Africa found some benefit of the intervention treatment on nutritional status or growth [17–20]. However, the effects of malaria prevention on the different measures of nutrition were inconsistent between these trials, making it difficult to draw any firm conclusions.

Given the possible direct effect of malaria on malnutrition it was investigated whether children who received a combination of the RTS,S/AS01_E_ malaria vaccine and SMC experienced a lower prevalence of acute or chronic malnutrition than children who received only one of the interventions.

## Methods

### Study sites

The RTS,S/AS01_E_ + SMC study was conducted in Bougouni and Ouelessebougou districts, Mali and in Houndé district, Burkina Faso. The climate of these areas is characterized by a rainy season from June to October, followed by a long dry season. Malaria, due predominantly to *Plasmodium falciparum*, is highly seasonal in both districts. The prevalence of *P. falciparum* malaria in school-age children was 22.5% in the study areas in Mali in November 2019 and 61.5% in the study area in Burkina Faso in December 2019 [5]. The UNICEF, WHO and World Bank joint global database on child malnutrition estimates the prevalence of stunting in children under five years of age at 23.8% in Burkina Faso and 26.4% in Mali, and the prevalence of wasting at 8.1% in Burkina Faso and 9.3% in Mali [21].

### Enrolment and randomization

All households within the study areas with children who would be 5–17 months old on April 1st 2017 were enumerated in February–March 2017. After written, informed consent had been obtained from parents or guardians, eligible children were allocated randomly to SMC alone, RTS,S/AS01_E_ alone, or SMC plus RTS,S/AS01_E_ combined groups by an independent statistician. The randomization list in each country used permuted blocks after sorting by age, gender, area of residence and prior receipt of SMC.

### Interventions

**Fig. 1:**
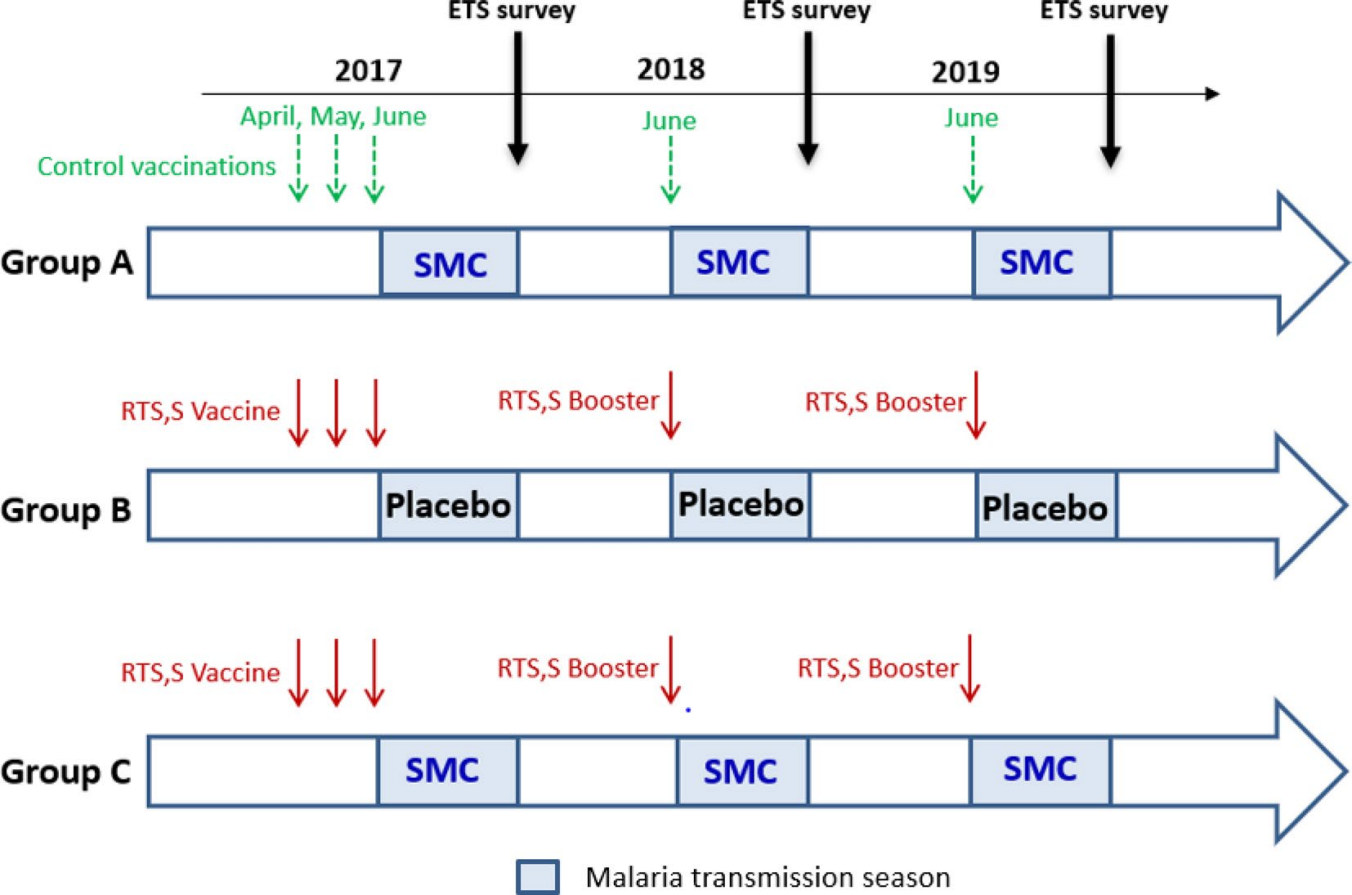
Study design. ETS survey, end of transmission season survey. Adapted from Chandramohan et al. [5]

Children in the RTS,S/AS01_E_ alone or RTS,S/AS01_E_ + SMC combined group received three injections of RTS,S/AS01_E_ vaccine (GSK, Rixensart, Belgium) at monthly intervals in April–June 2017 followed by fourth and fifth doses in June 2018 and June 2019, just prior to the malaria transmission season (Fig. 1). Children in the SMC alone group received three injections of rabies vaccine (*Rabipur*^*R*^) (Bavarian Nordic A/S, Denmark) in 2017 and one injection of Hepatitis A vaccine (HAVRIX^R)^ (GlaxoSmithkline, Rixensart, Belgium) in 2018 and 2019. The SMC alone and the combined group received four cycles of SMC at monthly intervals each year; the RTS,S/AS01_E_ alone group received four cycles of SMC placebo at the same times. A cycle of SMC for children aged ≥ 12 months comprised sulfadoxine/pyrimethamine 500/25 mg (SP) and amodiaquine (AQ) (Guilin Pharmaceuticals, Shanghai) 150 mg on day 1, and AQ 150 mg on days 2 and 3. Infants received half of these doses. All courses were administered by project staff under direct observation. All study children were given an insecticide-treated bed net at enrolment in 2017. Clinical episodes of malaria were detected by trial staff based at trial health facilities who tested children with suspected malaria with the use of a rapid diagnostic test. Children who had a positive result were treated with artemether–lumefantrine, and a blood film was obtained for subsequent microscopic examination (further details in Chandramohan et al. [5]). Serious adverse events were reported within 72 h after identification. Assignment of the causes of hospital admissions or deaths that occurred inside or outside the hospital was performed by two physicians who were unaware of the trial group assignments. A third independent physician reviewed cases for which there was a disagreement, and a consensus was reached.

### Study design

The analyses presented here use data collected at cross-sectional surveys during the course of the three-year trial. The cross-sectional surveys were conducted at the end of each malaria transmission season, approximately four weeks after the last SMC course in 2017, 2018 and 2019. All study children were invited to attend the end of transmission season surveys at trial health facilities or at fixed points in the community. See Fig. 1 (Adapted from Chandramohan et al. [5]). The incidence of passively-detected malnutrition outcomes over the course of the trial was also analysed as described below.

### Anthropometric measurements

During the cross-sectional surveys weight, height, and mid-upper arm circumference (MUAC) were measured by teams of trained nurses and field workers, supervised by physicians and nurses. All staff involved in taking the measurements were trained by senior trial clinicians on anthropometric measurement based on a Standard Operating Procedure before the survey. These staff were re-trained each year before the surveys. Children were weighed with their clothes on using a weighing scale (Seca©, Omron©, Constant©, Tanita©) that was calibrated with a standard weight at the start of each day; a sling and Salter scale were used for small children or the difference was calculated by weighing a mother and baby relative to the mother alone. Weight was recorded to the nearest 0.1 kg. Recumbent length measurements were taken for children under 2 years and standing height measurements for children above 2 years using a stadiometer (Seca©). MUAC was measured on the left arm to the nearest 0.1 cm with a MUAC tape. At the cross-sectional survey sites, specific stations were set-up for taking the measurements, with the scales and stadiometers placed on a flat surface. In general, each team used the same measuring instruments throughout the survey.

### Statistical analyses

Individual height-for-age (HAZ), weight-for-age (WAZ), weight-for-height (WHZ) and MUAC-for-age z-scores were calculated in Stata (Version 16, College Station, Texas) using the zanthro package with the WHO 2007 reference populations [22]. Outliers in weight, height and MUAC, and implausible z-scores identified using the current WHO recommended criteria [23], were excluded from the analysis. All cleaning and processing of nutritional data was performed blind to the child’s randomization group. The binary outcomes stunted, underweight, wasted and low MUAC-for-age were generated for each of the three study years, using the cleaned data and defined as individuals with corresponding z-scores below − 2 (HAZ, WAZ, WHZ, MUAC-for-age, respectively). The binary outcomes severely stunted, severely underweight, severely wasted and very low MUAC-for-age were also generated for each year, defined as individuals with corresponding z-scores below − 3. Individual changes in height, weight and MUAC were also calculated. While malnutrition can refer to both over- and under-nutrition, in this study, the term malnutrition will be used to refer only to under-nutrition, specifically stunting, wasting, underweight and low MUAC-for-age.

Each of the binary outcomes described above was compared between the three trial arms using Poisson regression with robust standard errors to obtain prevalence ratios [24]. As SMC is the current standard of care, the SMC alone group was considered as the reference group for comparisons with the combined and RTS,S/AS01_E_ alone groups. However, comparisons were also made between the combined and RTS,S/AS01_E_ alone groups by using the RTS,S/AS01_E_ alone group as the reference group. The primary outcome for acute malnutrition, pre-specified in the analytical plan, was prevalence of wasting in 2017, as at this point the study children were aged between 11 and 27 months, and within the age range in which the burden of acute malnutrition is the highest. As pre-specified in the analytical plan, the prevalence of wasting in 2018 is also of particular interest as study children would be aged between 23 and 39 months, the age range with a higher burden of malaria, therefore giving more scope for a malaria-specific intervention to exert effects on nutritional status. The primary outcome for chronic malnutrition, also pre-specified in the analytical plan, was prevalence of stunting in 2019, as at this point the study children had received the different interventions for three years, and differences in chronic malnutrition would be expected to be more apparent at this time.

In addition to the comparison of binary outcomes, linear regression was used to compare the mean z-scores and mean changes in anthropometric measures between the study arms, considering SMC as the reference group.

With a sample size of approximately 2000 children in each group at each of the cross-sectional surveys, assuming a prevalence of wasting of 15% and a prevalence of stunting of 20%, the study had more than 90% power to detect a 25% reduction in the prevalence of wasting in 2017. Similarly, the study had more than 90% power to detect a 25% reduction in the prevalence of stunting in 2019.

The primary analysis was a modified intention-to-treat (mITT) analysis (i.e., children who had received at least one dose of study vaccine) performed on the pooled data from both countries, controlling for country. As a secondary analysis, the two main primary outcomes were also analysed per protocol (defined as children who had received all scheduled vaccines in a specific year, and attended all the SMC contacts in that year), as pre-specified in the analytical plan.

The incidence of malnutrition requiring admission to hospital during the study period and clinically-diagnosed outpatient cases of malnutrition (where malnutrition was recorded as the diagnosis or main reason for care-seeking by the clinician) were compared between the study arms. The Andersen-Gill extension of the Cox regression model with a robust standard error to account for multiple episodes in the same child was used to estimate the hazard ratio and its 95% confidence interval comparing RTS,S/AS01_E_ alone and the combined intervention group to SMC alone, and comparing the combined group to RTS,S/AS01_E_ alone.

### Ethics and trial oversight

The trial protocol [25] was approved by the ethics committees of the London School of Hygiene & Tropical Medicine; the Ministry of Health, Burkina Faso; the University of Science, Techniques and Technologies of Bamako and by the national regulatory authorities of Burkina Faso and Mali. The Data Safety Monitoring Board (DSMB) reviewed serious adverse events, approved the statistical analysis plan and archived the locked databases prior to unblinding. A steering committee gave scientific advice and monitored progress of the trial. Written, informed consent was obtained from the parents or guardians of all children in the trial.

## Results

### Study population

**Fig. 2:**
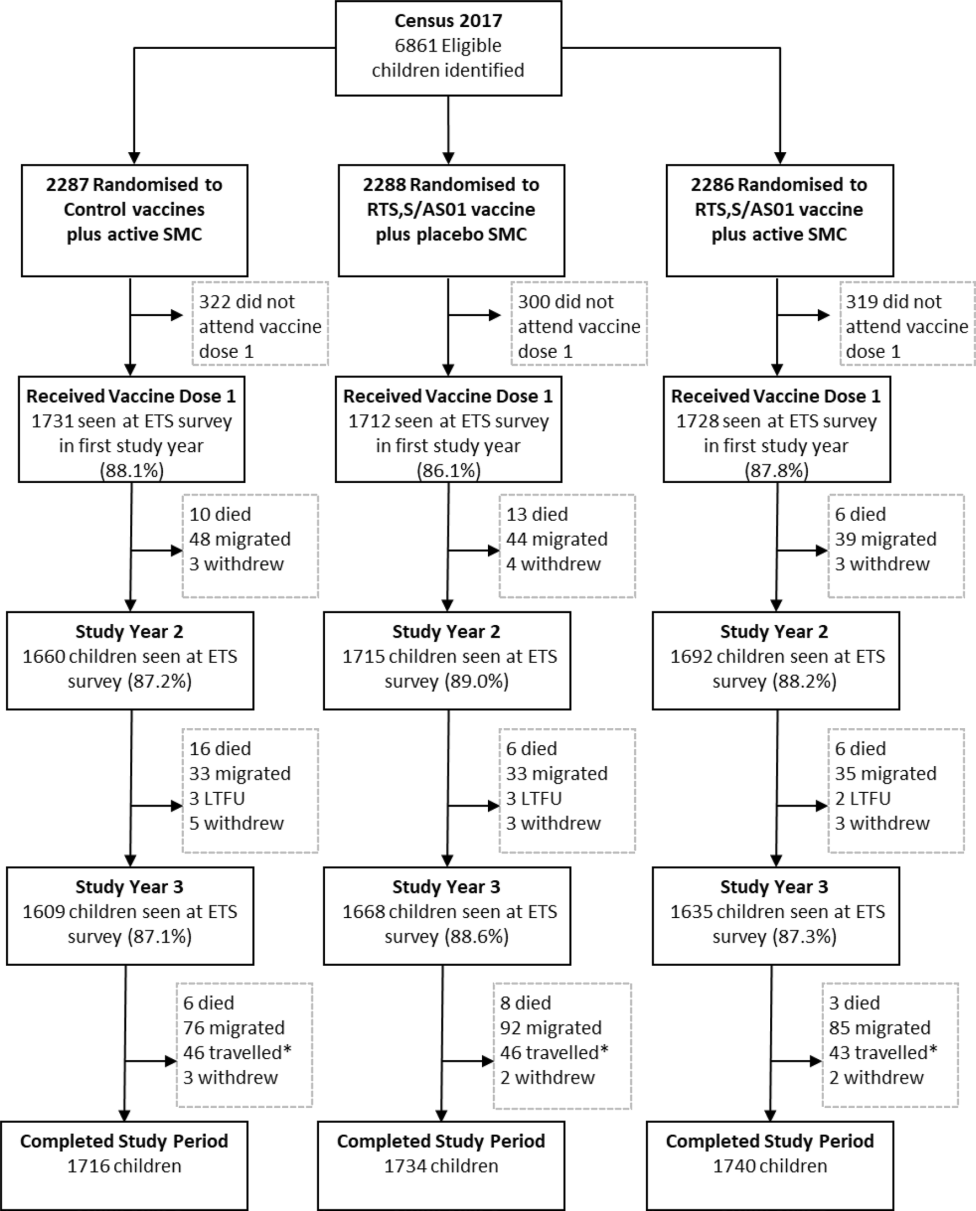
CONSORT Chart. Children who did not attend the first intervention contact (vaccine dose 1) were considered to have refused to participate in the trial. ETS, End of Transmission Season Survey; LFTU, lost-to-follow-up. All children that were seen at the ETS survey had their weight, height and MUAC recorded. *travelled denotes children who had temporarily travelled away from the study area at the time of the exit census but who had not permanently migrated. Adapted from Chandramohan et al. [5]

A total of 5920 children were enrolled in the study, of whom 1716 (87.3%), 1734 (87.2%) and 1740 (88.5%) children in the SMC alone, RTS,S/AS01_E_ alone or combined groups respectively, had completed their follow-up in March 2020 (Fig. 2). Out of the 5920 children enrolled in the study, 5171 (87.4%), 5067 (88.1%) and 4912 (87.7%) were seen at the 2017, 2018 and 2019 end of transmission season surveys, respectively (Table 1).

**Table 1:** Characteristics of the study children seen at the end of the malaria transmission season surveys by study arm

	SMC alone	RTS,S/AS01_E_ alone	RTS,S/AS01_E_ + SMC combined	Total
2017				
Number of children n/N (%)	1710/1965 (87.0)	1742/1988 (87.6)	1719/1967 (87.4)	5171/5920 (87.4)
Mean age (range) (months)	19 (11–27)	19 (12–27)	19 (12–27)	19 (11–27)
Sex n(%)				
Male	885 (51.8)	900 (51.7)	896 (52.1)	2681 (51.8)
Female	825 (48.2)	842 (48.3)	823 (47.9)	2490 (48.2)
2018				
Number of children n (%)	1655/1904 (86.9)	1717/1927 (89.1)	1695/1919 (88.3)	5067/5750 (88.1)
Mean age (range) (months)	31 (23–39)	31 (24–39)	31 (24–39)	31 (23–39)
Sex n (%)				
Male	844 (51.0)	898 (52.3)	863 (50.9)	2605 (51.4)
Female	811 (49.0)	819 (47.7)	832 (49.1)	2462 (48.6)
2019				
Number of children n (%)	1620/1847 (87.7)	1650/1882 (87.7)	1642/1873 (87.7)	4912/5602 (87.7)
Mean age (range) (months)	43 (35–50)	43 (36–50)	43 (36–50)	43 (35–50)
Sex n (%)				
Male	843 (52.0)	851 (51.6)	842 (51.3)	2536 (51.6)
Female	776 (47.9)	799 (48.4)	800 (48.7)	2375 (48.4)

### Changes in nutritional status over time by country

**Fig. 3:**
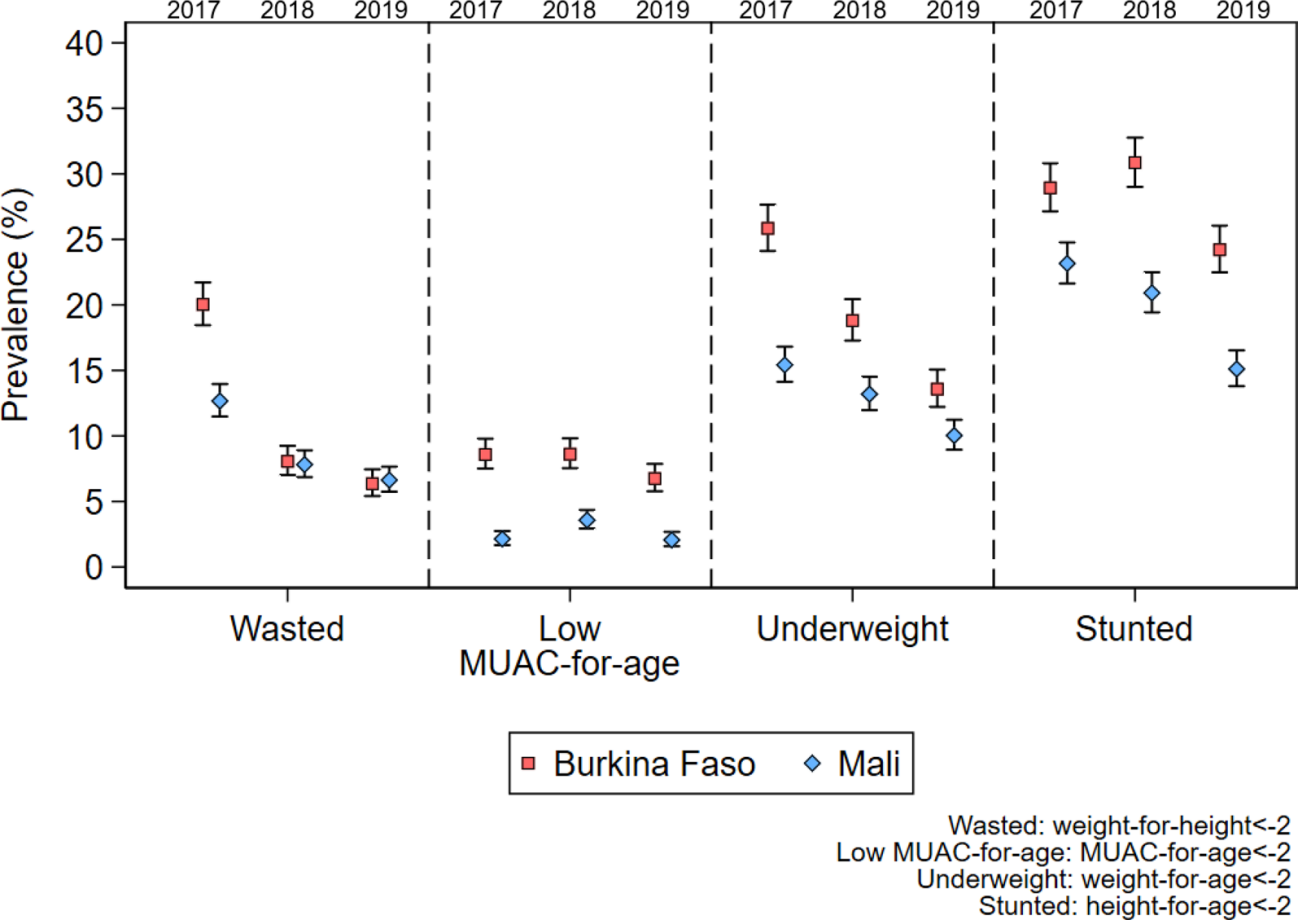
Prevalence of key nutritional outcomes in study children in Burkina Faso and Mali over the study period 2017–2019

Across the three trial groups, the prevalence of malnutrition was generally higher in Burkina Faso than in Mali, but decreased in both countries over the study period (Fig. 3). In Burkina Faso, the prevalence of wasting (WHZ < − 2) decreased from 20.0% (95% CI 18.4, 21.7) in 2017, when the children were 11–27 months old, to 6.4% (95% CI 5.4, 7.5) in 2019, when the children were 35–51 months old, while in Mali the corresponding estimates were 12.7% (95% CI 11.5, 14.0) in 2017 and 6.6% (95% CI 5.71, 7.65) in 2019. The prevalence of low MUAC-for-age (MUAC-for-age z-score < − 2) decreased slightly in Burkina Faso over the study period, falling from 8.6% (95% CI 7.5, 9.8) to 6.8% (95% CI 5.7, 7.9) but remained steady at around 2–3% over the study period in Mali. In Burkina Faso, the prevalence of being underweight (WAZ < − 2) or stunted (HAZ < − 2) decreased over the study period from 25.8% (95% CI 24.1, 27.7) to 13.6% (95% CI 12.2, 15.1) and from 28.9% (95% CI 27.1, 30.8) to 24.2% (95% CI 22.5, 26.1) respectively. In Mali, the prevalence of being underweight or stunted decreased from 15.4% (95% CI 14.2, 16.8) to 10.0% (95% CI 8.9, 11.2) and from 23.2% (95% CI 21.6, 24.8) to 15.1% (95% CI 13.8, 16.5) respectively. The prevalence of severe nutritional outcomes also decreased over the study period in both Mali and Burkina Faso (Additional file 1: Fig. S1).

### Effect of intervention group on nutritional outcomes

#### Acute malnutrition

**Fig. 4:**
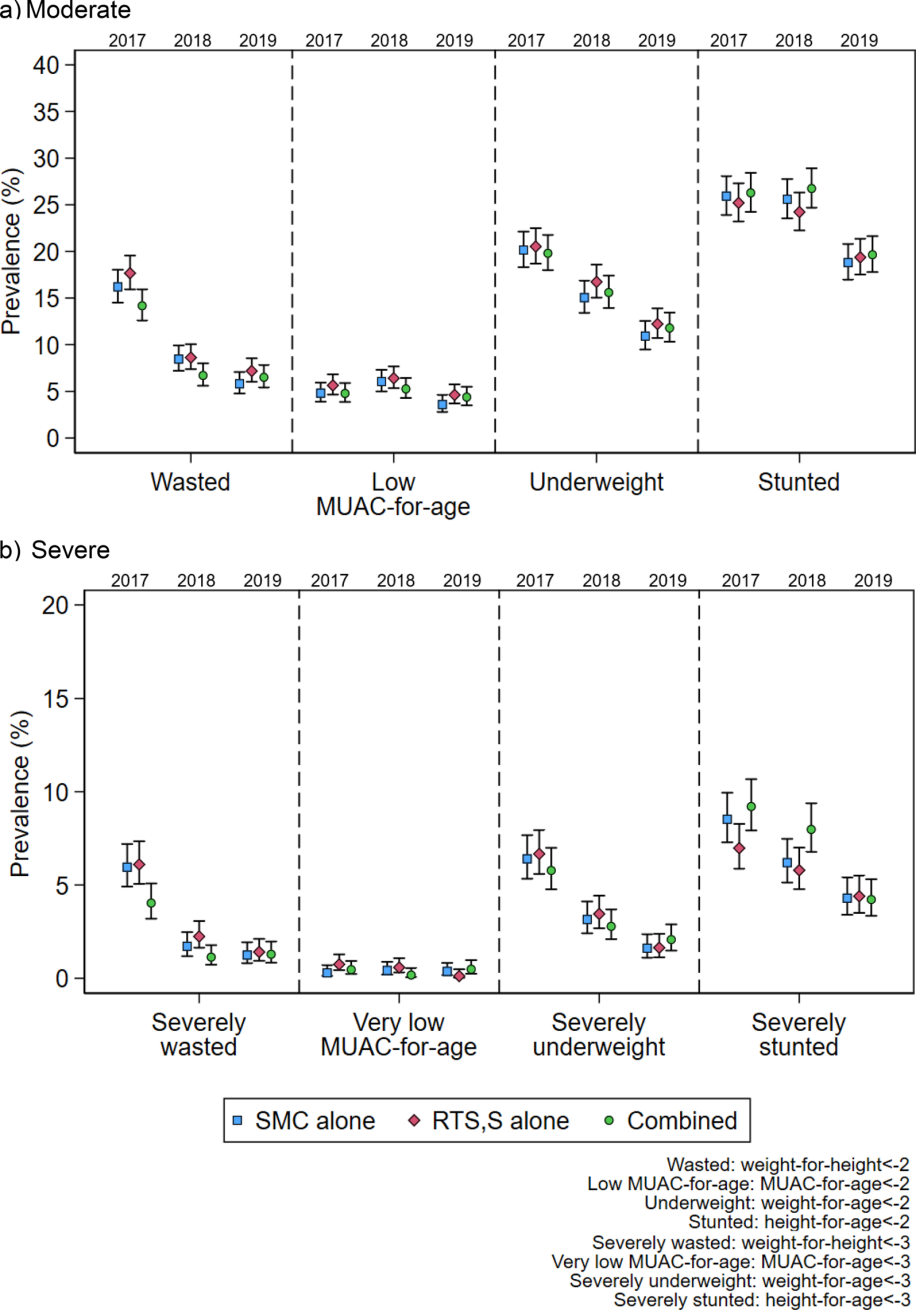
Prevalence of moderate (**a**) and severe (**b**) nutritional outcomes in study children between study arms (SMC, RTS,S/AS01_E_ and combined SMC + RTS,S/AS01) in Burkina Faso and Mali over the study period 2017–2019

The combined SMC plus RTS,S/AS01_E_ group had a lower prevalence of wasting than the other two groups in 2017 and 2018 (Table 2 and Fig. 4a). In the mITT analysis, the reduction in prevalence in wasting in the combined group was 12% compared to the SMC alone group in 2017 [PR 0.88 (0.75, 1.03)], and 20% compared to the RTS,S/AS01_E_ alone group [PR 0.80 (95% CI 0.69, 0.94)]. In 2018, the combined group had approximately 20% lower prevalence of wasting compared to both the SMC and RTS,S/AS01_E_ groups [PRs 0.79 (95% CI 0.62, 1.01) and 0.78 (95% CI 0.61, 0.99) respectively] (Table 2). Consistent with these findings, there was some evidence of a lower prevalence of severe wasting in the combined group compared to the other two groups in 2017 and 2018 (Table 2 and Fig. 4b). In both 2017 and 2018, the point estimate for the reduction in severe wasting in the combined group was larger than for moderate wasting, at least 32% relative to the SMC and RTS,S/AS01_E_ alone groups, but the confidence intervals were wider given the smaller number of children with severe wasting (Table 2). There was no evidence of a difference in the prevalence of moderate or severe wasting between the three groups in 2019.

**Table 2:** Prevalence and prevalence ratios of moderate and severe nutritional outcomes in study children between study arms in Burkina Faso and Mali at the end of the malaria transmission season surveys (mITT population)

	Group	n/N(%)	Prevalence Ratio (95% CI) RTS,S alone or combined group vs. SMC alone	Prevalence Ratio (95% CI) combined vs. RTS,S alone
Wasted^a^				
2017	SMC alone	272/1679 (16.2)	Reference	
	RTS,S alone	301/1704 (17.7)	1.10 (0.95, 1.27)	Reference
	Combined	239/1686 (14.2)	0.88 (0.75, 1.03)	0.80 (0.69, 0.94)
2018	SMC alone	138/1631 (8.46)	Reference	
	RTS,S alone	146/1693 (8.62)	1.02 (0.82, 1.27)	Reference
	Combined	112/1670 (6.71)	0.79 (0.62, 1.01)	0.78 (0.61, 0.99)
2019	SMC alone	93/1600 (5.81)	Reference	
	RTS,S alone	117/1629 (7.18)	1.23 (0.95, 1.61)	Reference
	Combined	106/1629 (6.51)	1.12 (0.85, 1.47)	0.91 (0.70, 1.17)
Severely wasted				
2017	SMC alone	100/1679 (5.96)	Reference	
	RTS,S alone	104/1704 (6.10)	1.04 (0.79, 1.35)	Reference
	Combined	68/1686 (4.03)	0.68 (0.51, 0.92)	0.66 (0.49, 0.89)
2018	SMC alone	28/1631 (1.72)	Reference	
	RTS,S alone	38/1693 (2.24)	1.30 (0.80, 2.11)	Reference
	Combined	19/1670 (1.14)	0.66 (0.37, 1.18)	0.51 (0.29, 0.88)
2019	SMC alone	20/1600 (1.25)	Reference	
	RTS,S alone	23/1629 (1.41)	1.12 (0.62, 2.03)	Reference
	Combined	21/1629 (1.29)	1.03 (0.56, 1.89)	0.92 (0.51, 1.65)
Low MUAC-for-age^b^				
2017	SMC alone	82/1706 (4.81)	Reference	
	RTS,S alone	98/1738 (5.64)	1.20 (0.90, 1.59)	Reference
	Combined	82/1717 (4.78)	1.01 (0.75, 1.36)	0.84 (0.64, 1.12)
2018	SMC alone	100/1653 (6.05)	Reference	
	RTS,S alone	110/1717 (6.41)	1.07 (0.82, 1.39)	Reference
	Combined	89/1692 (5.26)	0.87 (0.66, 1.15)	0.81 (0.62, 1.06)
2019	SMC alone	58/1617 (3.59)	Reference	
	RTS,S alone	76/1646 (4.62)	1.32 (0.95, 1.84)	Reference
	Combined	72/1642 (4.38)	1.23 (0.88, 1.73)	0.93 (0.68, 1.28)
Very low MUAC-for-age				
2017	SMC alone	5/1706 (0.29)	Reference	
	RTS,S alone	13/1738 (0.75)	2.60 (0.93, 7.26)	Reference
	Combined	8/1717 (0.47)	1.61 (0.53, 4.91)	0.62 (0.26, 1.49)
2018	SMC alone	7/1653 (0.42)	Reference	
	RTS,S alone	10/1717 (0.58)	1.38 (0.53, 3.61)	Reference
	Combined	3/1692 (0.18)	0.42 (0.11, 1.62)	0.30 (0.08, 1.10)
2019	SMC alone	6/1617 (0.37)	Reference	
	RTS,S alone	2/1646 (0.12)	0.34 (0.07, 1.66)	Reference
	Combined	8/1642 (0.49)	1.33 (0.46, 3.81)	3.94 (0.84, 18.5)
Underweight^c^				
2017	SMC alone	343/1702 (20.2)	Reference	
	RTS,S alone	357/1739 (20.5)	1.03 (0.90, 1.17)	Reference
	Combined	339/1712 (19.8)	0.99 (0.87, 1.13)	0.96 (0.85, 1.10)
2018	SMC alone	248/1648 (15.0)	Reference	
	RTS,S alone	286/1709 (16.7)	1.12 (0.96, 1.31)	Reference
	Combined	263/1687 (15.6)	1.04 (0.88, 1.21)	0.93 (0.80, 1.08)
2019	SMC alone	176/1611 (10.9)	Reference	
	RTS,S alone	201/1645 (12.2)	1.13 (0.93, 1.36)	Reference
	Combined	193/1637 (11.8)	1.08 (0.89, 1.31)	0.96 (0.80, 1.16)
Severely underweight				
2017	SMC alone	109/1702 (6.40)	Reference	
	RTS,S alone	116/1739 (6.67)	1.05 (0.82, 1.35)	Reference
	Combined	99/1712 (5.78)	0.91 (0.70, 1.19)	0.87 (0.67, 1.12)
2018	SMC alone	52/1648 (3.16)	Reference	
	RTS,S alone	59/1709 (3.45)	1.10 (0.76, 1.58)	Reference
	Combined	47/1687 (2.79)	0.88 (0.60, 1.30)	0.80 (0.55, 1.17)
2019	SMC alone	26/1611 (1.61)	Reference	
	RTS,S alone	27/1645 (1.64)	1.03 (0.60, 1.75)	Reference
	Combined	34/1637 (2.08)	1.29 (0.78, 2.14)	1.26 (0.76, 2.07)
Stunted^d^				
2017	SMC alone	441/1701 (25.9)	Reference	
	RTS,S alone	437/1734 (25.2)	0.98 (0.87, 1.09)	Reference
	Combined	448/1705 (26.3)	1.02 (0.91, 1.14)	1.04 (0.93, 1.17)
2018	SMC alone	421/1645 (25.6)	Reference	
	RTS,S alone	414/1709 (24.2)	0.95 (0.85, 1.07)	Reference
	Combined	449/1679 (26.7)	1.04 (0.93, 1.17)	1.10 (0.98, 1.23)
2019	SMC alone	302/1606 (18.8)	Reference	
	RTS,S alone	317/1637 (19.4)	1.04 (0.90, 1.20)	Reference
	Combined	321/1634 (19.6)	1.05 (0.91, 1.21)	1.01 (0.88, 1.16)
Severely stunted				
2017	SMC alone	145/1701 (8.52)	Reference	
	RTS,S alone	121/1734 (6.98)	0.82 (0.65, 1.03)	Reference
	Combined	157/1705 (9.21)	1.08 (0.87, 1.34)	1.32 (1.05, 1.66)
2018	SMC alone	102/1645 (6.20)	Reference	
	RTS,S alone	99/1709 (5.79)	0.94 (0.72, 1.23)	Reference
	Combined	134/1679 (7.98)	1.29 (1.00, 1.65)	1.37 (1.07, 1.76)
2019	SMC alone	69/1606 (4.30)	Reference	
	RTS,S alone	72/1637 (4.40)	1.04 (0.75, 1.43)	Reference
	Combined	69/1634 (4.22)	0.99 (0.71, 1.37)	0.95 (0.69, 1.32)

^**a**^ WHZ data missing for 102 children in 2017, 73 in 2018, 54 in 2019

^b^MUAC-for-age z-score data missing for 10 children in 2017, 5 in 2018, 7 in 2019

^c^WAZ data missing for 18 children in 2017, 23 in 2018, 19 in 2019

^d^HAZ data missing for 31 children in 2017, 34 in 2018, 35 in 2019

For all three years, the point estimate for the mean WHZ value was slightly higher in the combined group compared to the RTS,S/AS01_E_ alone group (Additional file 1: Table S1). The SMC group had comparable mean z-scores to the combined group, and thus slightly higher mean z-scores than the RTS,S/AS01_E_ group. However, the differences in mean z-score were very small, with a maximum difference between the groups of 0.11. Inspection of the cumulative distribution function indicated that the difference in the mean WHZ between the SMC and RTS,S/AS01_E_ alone groups largely arose at z-scores above the threshold for wasting (Additional file 1: Fig. S2). This is consistent with the results from the Poisson regression analysis that there was no difference between these groups in the prevalence of wasting (Table 2).

The results for the prevalence of low and very low MUAC-for-age differed from those observed for wasting; there was no evidence of a difference between the groups in the prevalence of low or very low MUAC-for-age for any of the years (Table 2 and Fig. 4). For mean MUAC-for-age z-score, as for mean WHZ, the combined and SMC groups had slightly higher point estimates compared to the RTS,S/AS01_E_ alone group, but again the differences were very small and largely arose above the threshold value for low-MUAC-for-age (Additional file 1: Table S1 and Fig. S2).

#### Chronic malnutrition

No marked difference in the prevalence of moderate stunting was found between any of the groups for any of the years, including the primary outcome, prevalence of stunting in 2019 (Table 2). Additionally, there was no difference in mean HAZ between the three groups for any of the years (Additional file 1: Table S1). However, there was some evidence of an increased prevalence of severe stunting in the combined group compared to the RTS,S/AS01_E_ alone group in 2017 [PR 1.32 (1.05, 1.66)], and in the combined group compared to both the SMC and RTS,S/AS01_E_ alone groups in 2018 [PRs 1.29 (95% CI 1.00, 1.65), and 1.37 (95% CI 1.07, 1.76), respectively] (Table 2).

There was no evidence of any differences between the three groups in the mean changes in weight, height and MUAC between the study years (Additional file 1: Table S2). No evidence was found for effect modification by country for the outcomes moderate or severe wasting and stunting in any year of the study (all P-values ≥ 0.15) (Additional file 1: Table S3 and Fig. S4).

#### Underweight

The prevalence ratios for moderately and severely underweight fluctuated around one for all comparisons across all three years, indicating no difference in the prevalence of being underweight between the three groups (Table 2). Similarly, in 2017 and 2018 there was no evidence of a difference in mean WAZ between the three groups. However, in 2019, there was evidence that the RTS,S/AS01_E_ alone group had a slightly lower mean WAZ than the SMC alone group [difference in mean WAZ: -0.073 (95% CI − 0.139, − 0.008)] (Additional file 1: Table S1).

Results of the per-protocol analysis for the primary outcomes were generally consistent with those from the mITT analysis (Additional file 1: Table S4).

### Incidence of hospitalization and clinically-diagnosed malnutrition

Over the three study years, 10 children were hospitalized due to a primary diagnosis of malnutrition, two in the SMC alone group, 3 in the RTS,S/AS01_E_ alone group, and five in the combined group (Table 3). In addition to the 10 children admitted to hospital with a primary diagnosis of malnutrition, an exploratory analysis of 18 children admitted to hospital with any malnutrition-related condition was conducted. This included 5 children with malnutrition as an immediate and underlying cause, 11 children where malnutrition was an underlying cause only, and 2 children where malnutrition was a contributory cause. Given the very small numbers, all the hazard ratios had wide confidence intervals which overlapped one. There were 148 cases of clinically-diagnosed malnutrition among study children over the three years. Overall, the incidence of clinically diagnosed malnutrition was slightly higher in the RTS,S/AS01_E_ alone group compared to the SMC alone and combined groups. This difference was largely driven by a higher incidence in the RTS,S/AS01_E_ alone group in year 2, where the incidence of clinical malnutrition was 9.98/1000 person years at risk (PYAR), compared to 4.82/1000 and 4.77/1000 in the SMC alone and combined groups, respectively. However, the confidence intervals were wide, reflecting the relatively small number of events, and all of the confidence intervals overlapped one.

**Table 3:** Incidence of hospitalized and clinically diagnosed malnutrition in study children by study group overall and by study year (mITT population)

Outcomes	PYAR	Events	Rate per 1000 PYAR (95% CI)	Hazard ratio (95% CI) RTS,S alone or combined groups vs. SMC alone	Hazard ratio (95% CI) Combined vs. RTS,S alone
Hospitalization for primary diagnosis of malnutrition					
SMC alone	5449.9	2	0.367 (0.092, 1.47)	Reference	
RTS,S alone	5535.7	3	0.542 (0.175, 1.68)	1.48 (0.25, 8.80)	Reference
Combined	5508.0	5	0.908 (0.378, 2.18)	2.48 (0.48, 12.8)	1.68 (0.40, 6.94)
Hospitalization for malnutrition-related conditions					
SMC alone	5449.9	5	0.92 (0.38, 2.20)	Reference	
RTS,S alone	5535.7	6	1.08 (0.49, 2.41)	1.20 (0.37, 3.91)	Reference
Combined	5508.0	7	1.27 (0.61, 2.67)	1.40 (0.44, 4.39)	1.16 (0.39, 3.44)
Clinically diagnosed malnutrition					
SMC alone	5449.9	49	8.99 (6.80, 11.9)	Reference	
RTS,S alone	5535.7	58	10.5 (8.10, 13.6)	1.14 (0.65, 1.99)	Reference
Combined	5508.0	41	7.44 (5.48, 10.1)	0.82 (0.49, 1.40)	0.73 (0.40, 1.30)
Year 1					
SMC alone	1794.3	39	21.7 (15.9, 29.7)	Reference	
RTS,S alone	1816.8	33	18.2 (12.9, 25.5)	0.81 (0.45, 1.46)	Reference
Combined	1802.3	29	16.1 (11.2, 23.2)	0.73 (0.41, 1.33)	0.90 (0.47, 1.72)
Year 2					
SMC alone	1868.5	9	4.82 (2.51, 9.26)	Reference	
RTS,S alone	1903.4	19	9.98 (6.37, 15.6)	2.03 (0.77, 5.31)	Reference
Combined	1894.4	9	4.75 (2.47, 9.13)	0.98 (0.37, 2.59)	0.48 (0.19, 1.21)
Year 3					
SMC alone	1787.1	1	0.560 (0.079, 3.97)	Reference	
RTS,S alone	1815.5	6	3.30 (1.48, 7.36)	5.73 (0.66, 50.0)	Reference
Combined	1811.3	3	1.66 (0.534, 5.14)	2.94 (0.31, 28.2)	0.51 (0.12, 2.21)

## Discussion

The trial in which the nutritional data presented in this paper were collected measured a large reduction in cases of uncomplicated and severe malaria in the combined RTS,S/AS01_E_ plus SMC group over the three-year study, compared to children who were randomized to receive only one of these interventions. Children in the trial who received the combined intervention experienced around 60% fewer cases of clinical malaria, and around 70% fewer hospital admissions with severe malaria, compared to children who only received SMC alone or RTS,S/AS01_E_ alone [5].

The prevalence of wasting was lower in the first two years of the study in the combined group than in the SMC alone or RTS,S/AS01_E_ alone groups. There was an approximately 20% reduction in the prevalence of moderate wasting and the point estimate for the reduction in the prevalence of severe wasting in the combined group was at least 30% compared to the SMC alone or RTS,S/AS01_E_ alone groups at the time of the end of malaria transmission season surveys in 2017 and 2018, when the study cohort was aged between 11 and 27 months and 23 and 39 months, respectively. However, no difference in the prevalence of moderate or severe wasting between the groups was found in 2019. This may be because there was a slightly lower overall prevalence of wasting in 2019, and the children had passed the age where there is typically the greatest burden of acute malnutrition. Additionally, it is possible that malaria is a less important cause of acute malnutrition in older children.

Acute malnutrition was also assessed through comparison of the prevalence of low or very low MUAC-for-age between the study groups, but there was no evidence of a difference in any of the years. The difference between the results for wasting and MUAC-for-age may be because MUAC is a less sensitive measure of acute malnutrition than weight-for-height, even when age is taken into account. There was some evidence of a difference in mean WHZ and MUAC-for-age z-score between the groups in all three years of the study, but the maximum difference for any of the comparisons was a difference in z-score of 0.12, which is not likely to be clinically relevant. No difference was found between the groups in mean weight or MUAC gain between the study years.

These results are in line with many previous malaria prevention randomized controlled trials that have found some effect of a reduction in malaria cases on acute nutritional status. However, there is a lack of consistency between these studies in terms of which specific measures of nutrition were affected by a reduction in malaria. For example, two trials of SMC in Boussé District, Burkina Faso and Kati District, Mali, with identical designs, observed some increased weight gain in children in the intervention group over one malaria transmission season, with one of the trials also finding evidence of a 21% (95% CI 0.65, 1.00) reduction in the risk of wasting and a 16% (95% CI 0.72, 0.99) reduction in the risk of being underweight in children who received SMC [17, 18]. Additionally, two trials in infants in Kenya found modest increased mean WAZ and MUAC-for-age z-score in infants who slept under a bed net [7, 15]. Furthermore, a community-randomized insecticide-treated bed net trial in Gambian children resulted in slightly higher mean WAZ and WHZ in intervention villages after one malaria transmission season [14]. Compared to the RTS,S/AS01_E_ plus SMC trial, these trials generally had much higher incidence rates of malaria in the control groups because in the current trial, all groups received SMC or RTS,S/AS01_E_ vaccine, or both, and were given bed nets. It is possible that malaria has a larger effect on acute malnutrition at higher incidence rates, when it is common for clinical malaria episodes to recur in a short time frame; this was not observed in our study because incidence rates were much lower.

This study found no evidence that the reduction in malaria in the combined group had a beneficial effect on stunting. This is consistent with a recent literature review of observational studies and randomized control trials of malaria interventions that found limited evidence for an effect of clinical malaria episodes or parasitaemia on stunting or growth velocity [26]. A common limitation amongst previous studies is that the duration of the studies, often conducted over only one malaria transmission season, may have been insufficient to detect the effect of malaria or malaria prevention interventions on stunting (a result of chronic malnutrition). In contrast, this study was conducted over a three-year period but, nevertheless, no impact on stunting was seen in children in the combined group compared with the children in the RTS,S/AS01_E_ or SMC alone groups.

While this study found no evidence of a difference between any of the groups in the prevalence of moderate stunting, mean height-for-age z-score, or mean gain in height, the point estimates for the prevalence of severe stunting were higher in the combined group compared to the RTS,S/AS01_E_ alone group in 2017, and compared to both single intervention groups in 2018. There is no obvious explanation for these results, particularly as there was no effect on moderate stunting in the same period and any non-specific effects of the interventions would have also likely been observed in the single intervention groups. This could be a chance finding given the number of comparisons undertaken. The repetition of this finding in the second year of the study (which would normally speak to plausibility of the association) may be because stunting is, by nature, a long-term condition. Thus, a chance difference in low height-for-age z-score in the combined group may have persisted over these two years.

Additionally, there was a modest increase in the incidence of clinically diagnosed malnutrition in the RTS,S/AS01_E_ alone group compared to the SMC alone and combined groups. It is possible that this difference arose through non-malaria effects of sulfadoxine-pyrimethamine (including its broader antimicrobial activity), a benefit that the RTS,S/AS01_E_ alone group would not receive, given the *P. falciparum*-specific effect of RTS,S/AS01_E_ [27, 28]. However, these differences were not consistent across the study years and this could be a chance finding, but may be worth further exploration in other studies.

Acute and chronic malnutrition at the end of the malaria transmission season were common in this cohort of children, especially in the first year of the study when the children were the youngest. The prevalence of stunting, wasting and being underweight were largely similar in this study to the national nutrition estimates when comparing data from children of a similar age [21, 29]. However, the prevalence of stunting and being underweight were roughly 5–10% lower in the study children in Mali than that reported in the 2018 Mali Demographic and Health Survey. This could be due to the fact that the participants in Mali came from a semi-urban area and, therefore, may not be representative of the national population, and could also be due to annual variations in the prevalence of malnutrition.

Though this was a large, well-powered study with a high prevalence of malnutrition and high incidence rate of malaria, a long follow-up period with high study retention and a large reduction in malaria-related outcomes in the combined group, the study had some limitations. The anthropometric measurements were only taken at one time point each year, at the end of the malaria transmission season. These measurements were taken during the period after the hunger season, where children gain weight following the harvest. It is, therefore, possible that the effect of the seasonal changes in acute malnutrition due to the hunger season are much larger than that of malaria infection, which may have hidden part of a smaller effect of malaria on acute malnutrition. Furthermore, it is possible that there could have been some measurement bias due to human error; while efforts were taken to ensure the anthropometric measurements were accurately recorded, there may have been some inaccuracy as the measurements were taken in large numbers of children in a field setting. Weighing children with their clothes on could have been an additional source of measurement error. Additionally, easy access to diagnosis and treatment of malaria in the study children when unwell, may have prevented prolonged infection and limited the potential effect of malaria on nutritional status.

## Conclusions

Despite a high prevalence of malnutrition and incidence of malaria in the study populations and a major reduction in the incidence of malaria in children receiving both interventions, this had only a modest impact on nutritional status that was not consistent across study years and different nutritional indices. Nutritional status among children in both countries was poor, and thus other interventions are needed to reduce the high burden of malnutrition seen in the study areas.

## Supplementary Information

**Additional file 1: Figure S1. **Prevalence of severe nutritional outcomes in study children Burkina Faso and Mali over the study period 2017-2019 (mITT population). **Table S1. **Difference in mean Z-scores for nutritional indicators between study arms in Burkina Faso and Mali at the end of the malaria transmission season surveys (mITT population).** Figure S2. **Cumulative distribution functions of weight-for-height and MUAC-for-age in study children between study arms over the study period 2017-2019, centred on the Z-score cut-off for moderate malnutrition (mITT population).** Figure S3**. Cumulative distribution functions of weight-for-age and height-for-age in study children between study arms over the study period 2017-2019, centred on the Z-score cut-off for moderate malnutrition (mITT population). **Table S2. **Difference in mean changes in anthropometric measurements between study arms in Burkina Faso and Mali at the end of transmission season surveys between 2017-2018, 2018-2019 and 2017-2019. **Table S3. **Test of interaction between country and treatment arm. **Figure S4. **Prevalence of key nutritional outcomes in study children between study arms (SMC, RTS,S/AS01_E_ and combined SMC + RTS,S/AS01_E_) in Burkina Faso (a) and Mali (b) over the study period 2017-2019 (mITT population). **Table S4. **Prevalence and prevalence ratios of primary outcomes between study arms in Burkina Faso and Mali at the end of the malaria transmission season surveys (per protocol population).

## Data Availability

The datasets generated and analysed during the current study will be archived on the LSHTM Data Compass system (http://datacompass.lshtm.ac.uk/) and assigned a Digital Object Identifier (DOI). A Data Access Group will review requests to share the archived data to ensure that the data are only used for appropriate research purposes and that participants’ privacy is maintained. In April 2020, study children completing the present trial were re‐enrolled into an extension study for two additional years. Therefore, data from the first three years of the study will be made available in November 2022, when the extension study has been completed.

## Abbreviations

ETS survey: End of transmission season survey
HAZ: Height-for-age z-score
LFTU: Lost-to-follow-up
mITT: Modified intention-to-treat
MUAC: Mid-upper arm circumference
PR: Prevalence ratio
PYAR: Person years at risk
SMC: Seasonal malaria chemoprevention
UNICEF: United Nations Children’s Fund
WAZ: Weight-for-age z-score
WHO: World Health Organization
WHZ: Weight-for-height z-score
