# Supplementary material for: Impact of seasonal RTS,S/AS01E vaccination plus seasonal malaria chemoprevention on the nutritional status of children in Burkina Faso and Mali
Source: Malar J. 2022 Feb 22;21:59. doi: 10.1186/s12936-022-04077-x (PMC8864823; doi:10.1186/s12936-022-04077-x)
Supplement: Supplementary file 1 — Additional file 1: Figure S1. Prevalence of severe nutritional outcomes in study children Burkina Faso and Mali over the study period 2017-2019 (mITT population). Table S1. Difference in mean Z-scores for nutritional indicators between study arms in Burkina Faso and Mali at the end of the malaria transmission season surveys (mITT population). Figure S2. Cumulative distribution functions of weight-for-height and MUAC-for-age in study children between study arms over the study period 2017-2019, centred on the Z-score cut-off for moderate malnutrition (mITT population). Figure S3. Cumulative distribution functions of weight-for-age and height-for-age in study children between study arms over the study period 2017-2019, centred on the Z-score cut-off for moderate malnutrition (mITT population). Table S2. Difference in mean changes in anthropometric measurements between study arms in Burkina Faso and Mali at the end of transmission season surveys between 2017-2018, 2018-2019 and 2017-2019. Table S3. Test of interaction between country and treatment arm. Figure S4. Prevalence of key nutritional outcomes in study children between study arms (SMC, RTS,S/AS01E and combined SMC + RTS,S/AS01E) in Burkina Faso (a) and Mali (b) over the study period 2017-2019 (mITT population). Table S4. Prevalence and prevalence ratios of primary outcomes between study arms in Burkina Faso and Mali at the end of the malaria transmission season surveys (per protocol population). [file 12936_2022_4077_MOESM1_ESM.docx]

**Additional file 1**

**Figure S1. Prevalence of severe nutritional outcomes in study children Burkina Faso and Mali over the study period 2017-2019 (mITT population)**


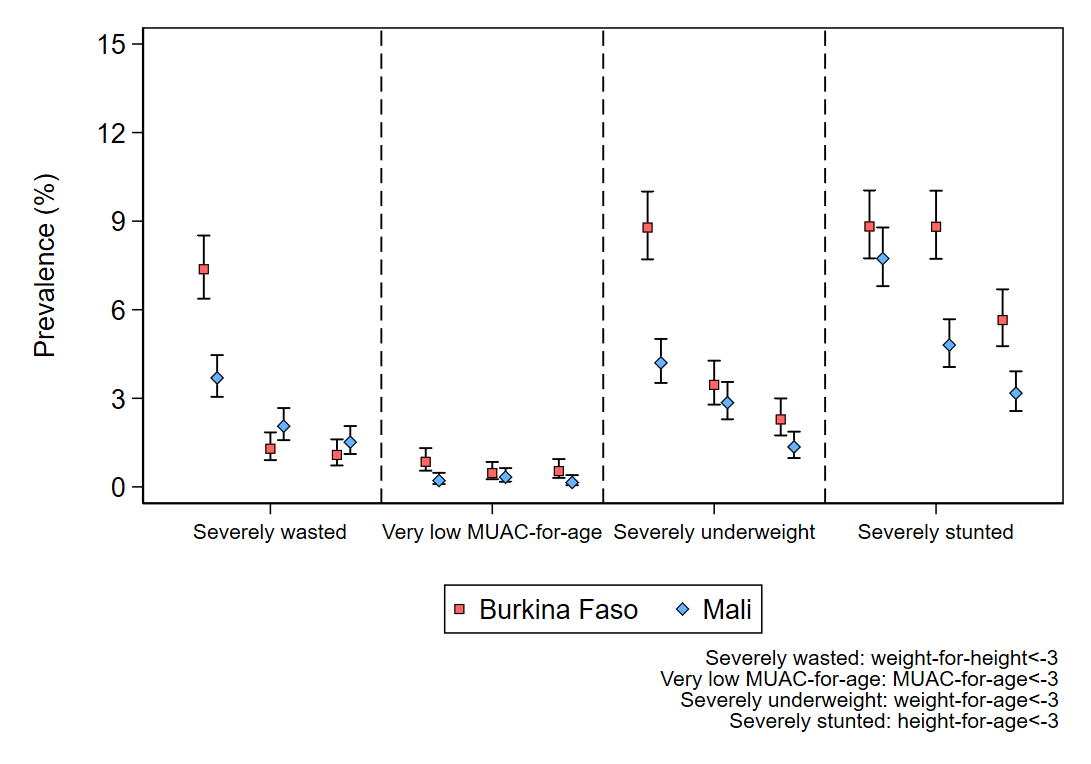

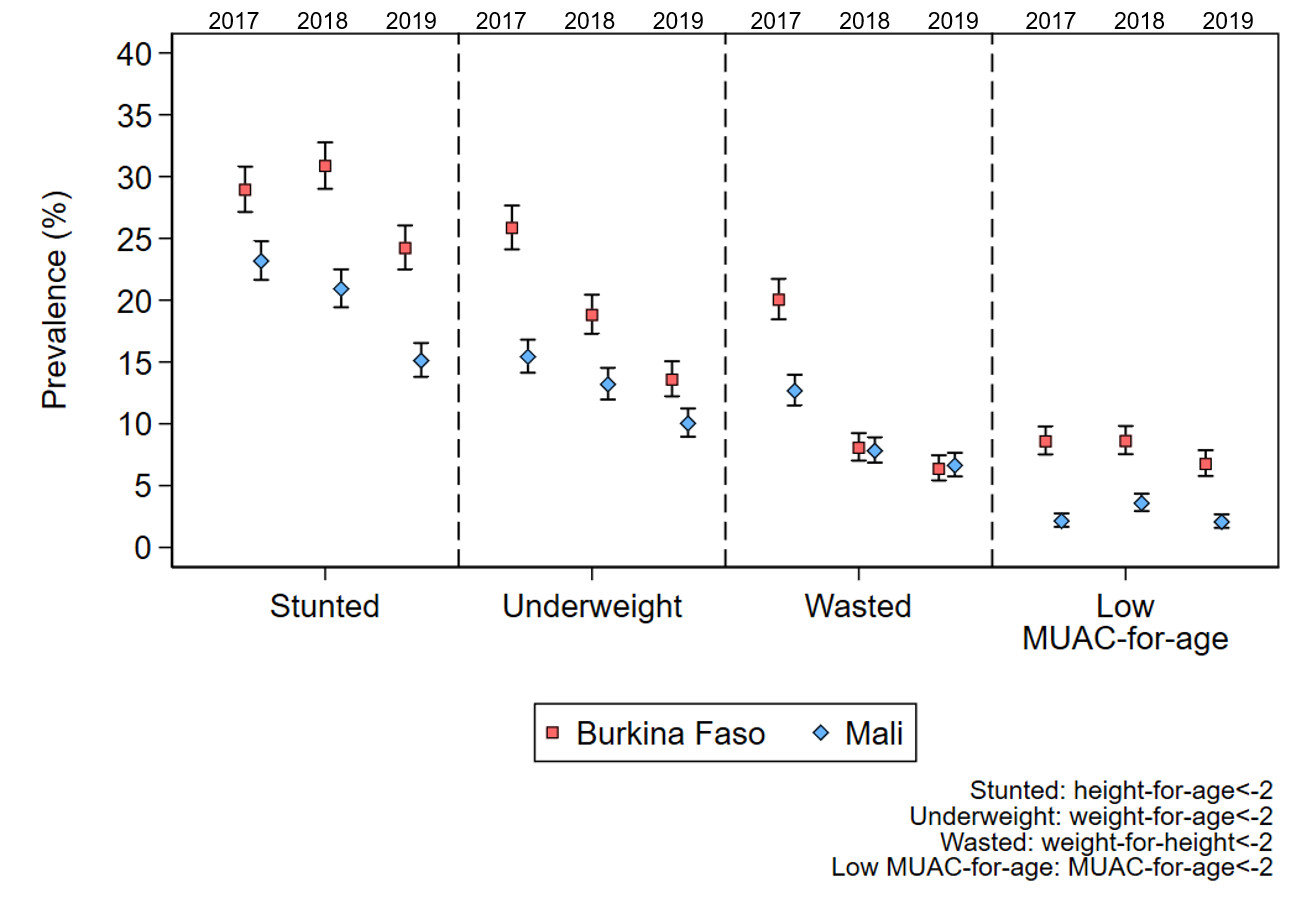


**Table S1. Difference in mean Z-scores for nutritional indicators between study arms in Burkina Faso and Mali at the end of the malaria transmission season surveys (mITT population)**

|  | **Group** | **Mean (SD), N** | **Difference in mean (95% CI) RTS,S alone or Combined group vs. SMC alone** | **Difference in mean (95% CI) Combined vs. RTS,S alone** |
| --- | --- | --- | --- | --- |
| **Weight-for-height (Z-score)** | | | | |
| 2017 | SMC alone | -0.779 (1.33), 1679 | Reference |  |
|  | RTS,S alone | -0.812 (1.33), 1704 | -0.039 (-0.126, 0.049) | Reference |
|  | Combined | -0.701 (1.28), 1686 | 0.073 (-0.015, 0.161) | 0.112 (0.024, 0.199) |
|  |  |  |  |  |
| 2018 | SMC alone | -0.396 (1.16), 1631 | Reference |  |
|  | RTS,S alone | -0.474 (1.16), 1693 | -0.081 (-0.158, -0.003) | Reference |
|  | Combined | -0.373 (1.12), 1670 | 0.022 (-0.056, 0.100) | 0.103 (0.026, 0.180) |
|  |  |  |  |  |
| 2019 | SMC alone | -0.326 (1.09), 1600 | Reference |  |
|  | RTS,S alone | -0.426 (1.06), 1629 | -0.100 (-0.174, -0.026) | Reference |
|  | Combined | -0.350 (1.06), 1629 | -0.024 (-0.098, 0.050) | 0.076 (0.002, 0.149) |
|  |  |  |  |  |
| **MUAC-for-age (Z-score)** | | | | |
| 2017 | SMC alone | -0.437 (0.97), 1706 | Reference |  |
|  | RTS,S alone | -0.529 (0.95), 1738 | -0.099 (-0.161, -0.037) | Reference |
|  | Combined | -0.459 (0.96), 1717 | -0.028 (-0.090, 0.034) | 0.071 (0.010, 0.133) |
|  |  |  |  |  |
| 2018 | SMC alone | -0.605 (0.90), 1653 | Reference |  |
|  | RTS,S alone | -0.689 (0.89), 1717 | -0.090 (-0.148, -0.032) | Reference |
|  | Combined | -0.596 (0.89), 1692 | 0.009 (-0.049, 0.068) | 0.099 (0.042, 0.157) |
|  |  |  |  |  |
| 2019 | SMC alone | -0.473 (0.86), 1617 | Reference |  |
|  | RTS,S alone | -0.578 (0.85), 1646 | -0.117 (-0.174, -0.060) | Reference |
|  | Combined | -0.504 (0.89), 1642 | -0.036 (-0.093, 0.021) | 0.081 (0.025, 0.138) |
|  |  |  |  |  |
| **Weight-for-age (Z-score)** | | | | |
| 2017 | SMC alone | -1.034 (1.27), 1702 | Reference |  |
|  | RTS,S alone | -1.070 (1.27), 1739 | -0.043 (-0.125, 0.040) | Reference |
|  | Combined | -1.008 (1.23), 1712 | 0.019 (-0.064, 0.102) | 0.062 (-0.021, 0.144) |
|  |  |  |  |  |
| 2018 | SMC alone | -0.947 (1.11), 1648 | Reference |  |
|  | RTS,S alone | -0.995 (1.06), 1709 | -0.053 (-0.125, 0.019) | Reference |
|  | Combined | -0.943 (1.07), 1687 | 0.004 (-0.068, 0.077) | 0.057 (-0.015, 0.129) |
|  |  |  |  |  |
| 2019 | SMC alone | -0.839 (0.95), 1611 | Reference |  |
|  | RTS,S alone | -0.907 (0.96), 1645 | -0.073 (-0.139, -0.008) | Reference |
|  | Combined | -0.868 (0.98), 1637 | -0.031 (-0.097, 0.034) | 0.042 (-0.023, 0.107) |
|  |  |  |  |  |
| **Height-for-age (Z-score)** | | | | |
| 2017 | SMC alone | -1.107 (1.49), 1701 | Reference |  |
|  | RTS,S alone | -1.084 (1.44), 1734 | 0.018 (-0.079, 0.115) | Reference |
|  | Combined | -1.155 (1.45), 1705 | -0.052 (-0.149, 0.046) | -0.070 (-0.166, 0.027) |
|  |  |  |  |  |
| 2018 | SMC alone | -1.203 (1.26), 1645 | Reference |  |
|  | RTS,S alone | -1.205 (1.21), 1709 | -0.006 (-0.089, 0.077) | Reference |
|  | Combined | -1.249 (1.24), 1679 | -0.045 (-0.128, 0.038) | -0.039 (-0.122, 0.043) |
|  |  |  |  |  |
| 2019 | SMC alone | -1.066 (1.14), 1606 | Reference |  |
|  | RTS,S alone | -1.049 (1.16), 1637 | 0.007 (-0.070, 0.085) | Reference |
|  | Combined | -1.084 (1.12), 1634 | -0.023 (-0.100, 0.054) | -0.030 (-0.107, 0.046) |

**Figure S2. Cumulative distribution functions of weight-for-height and MUAC-for-age in study children between study arms over the study period 2017-2019, centred on the Z-score cut-off for moderate malnutrition (mITT population)**

**2017**

**2018**


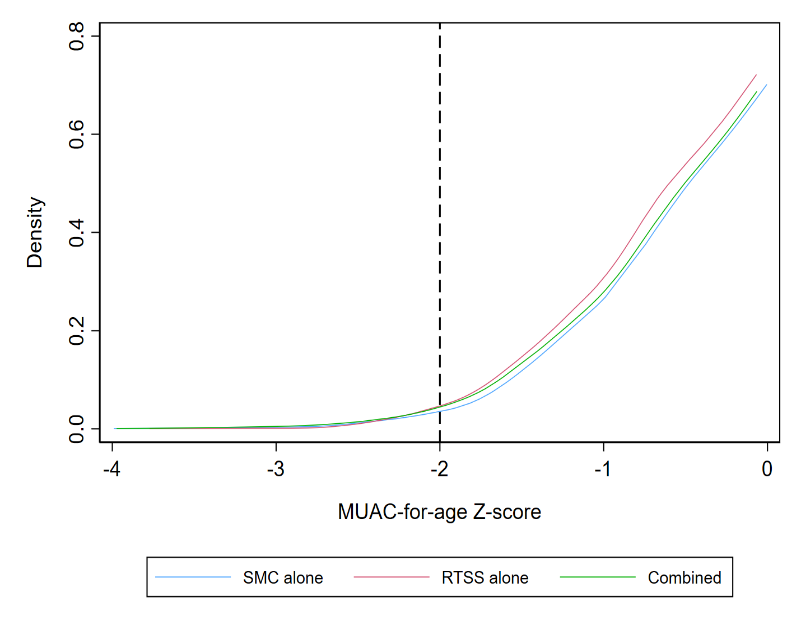

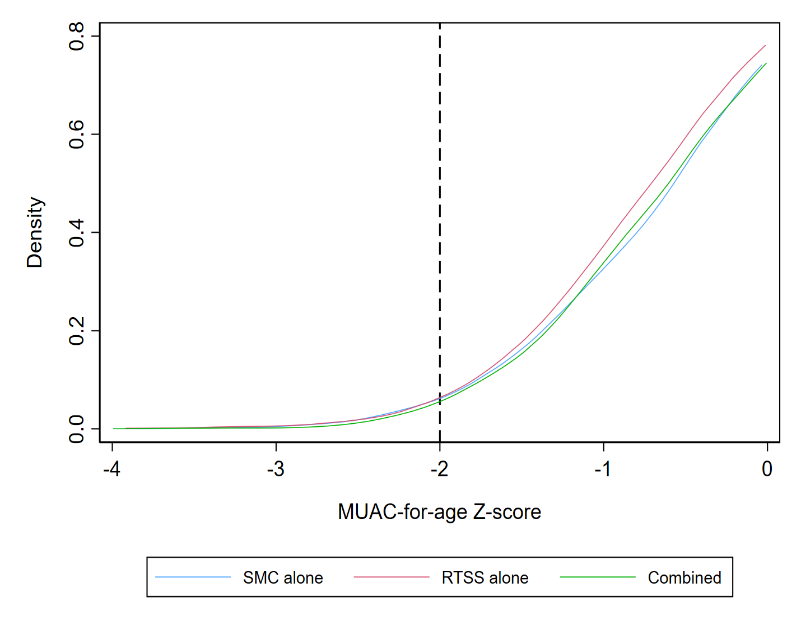

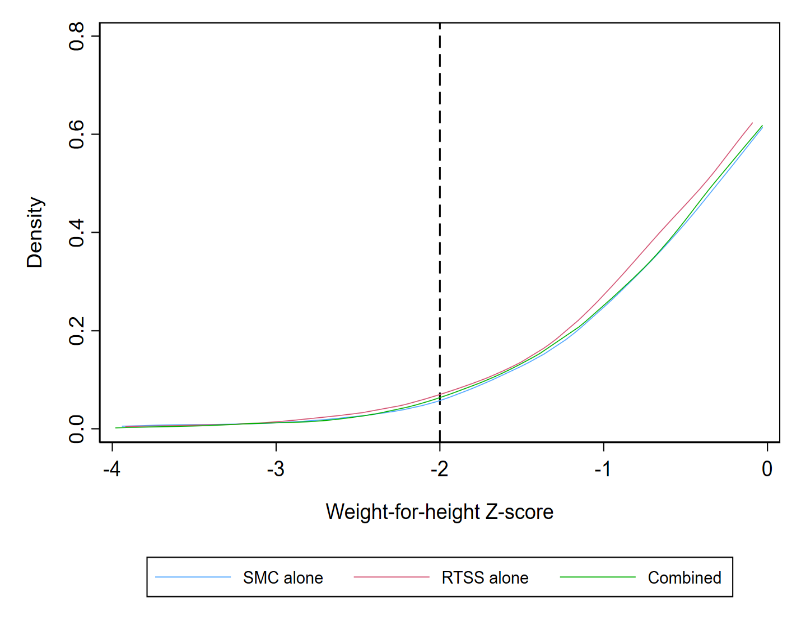

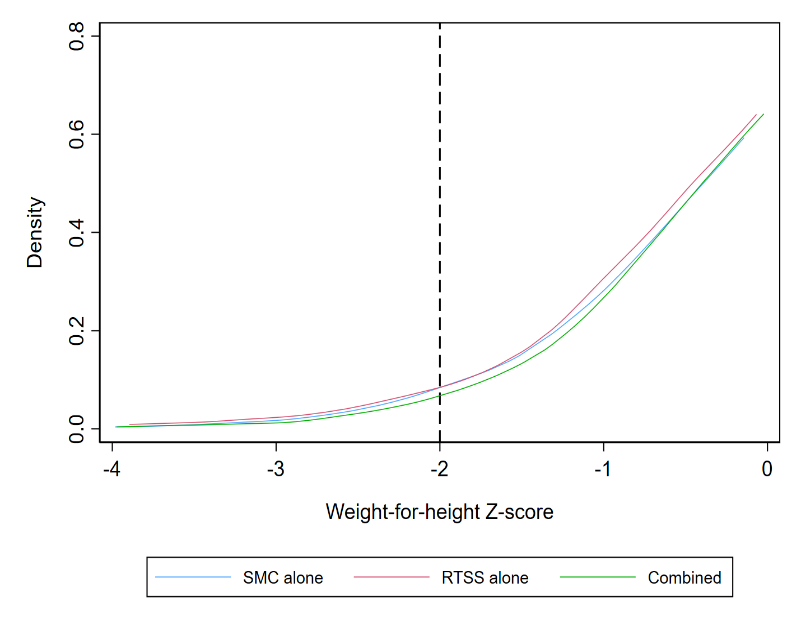

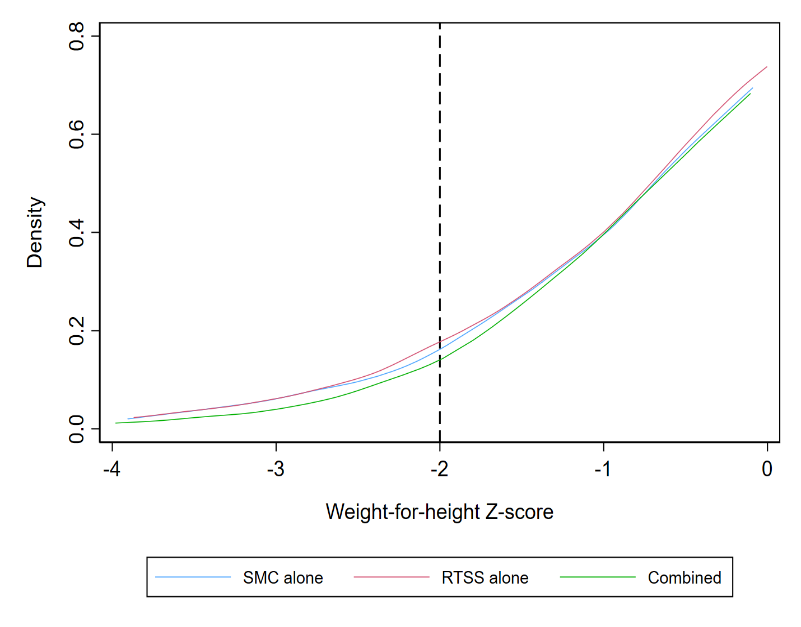

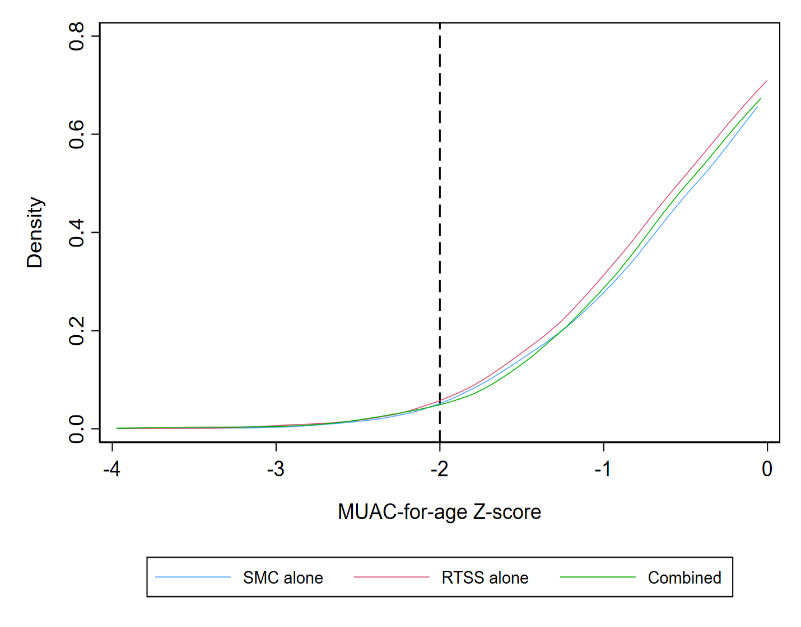

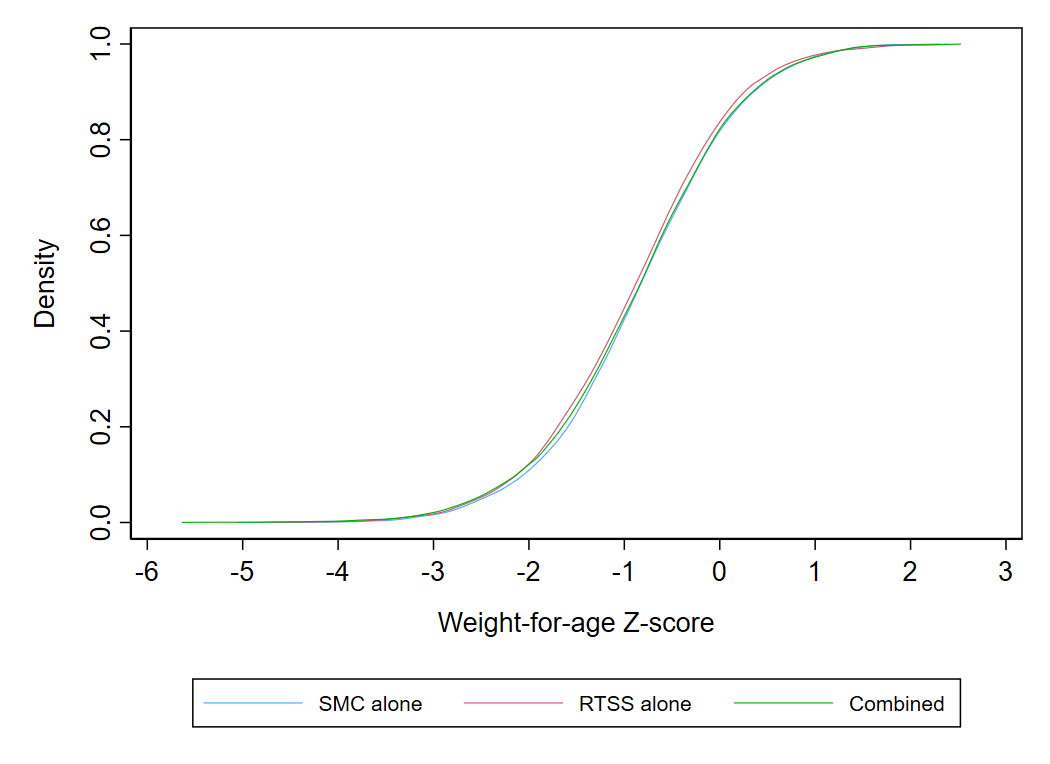


**2019**

**Figure S3. Cumulative distribution functions of weight-for-age and height-for-age in study children between study arms over the study period 2017-2019, centred on the Z-score cut-off for moderate malnutrition (mITT population)**

**2017**


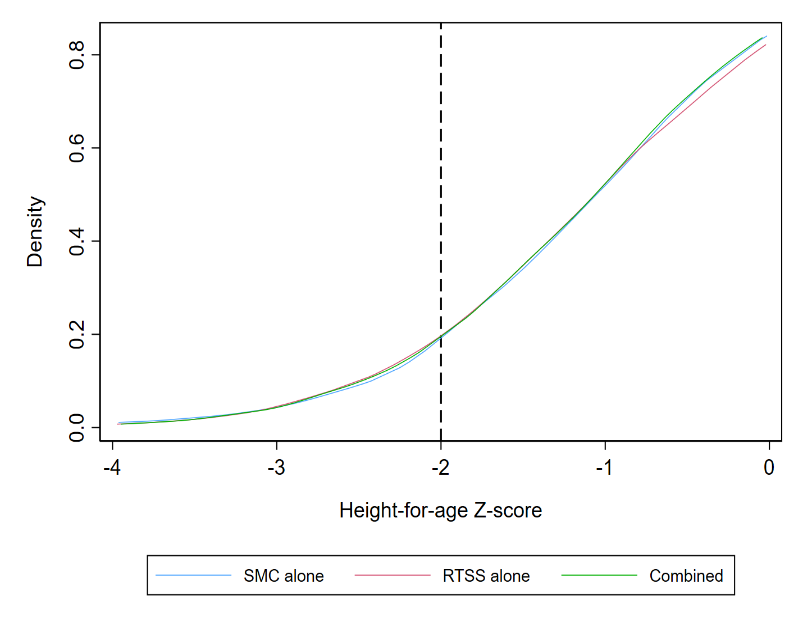

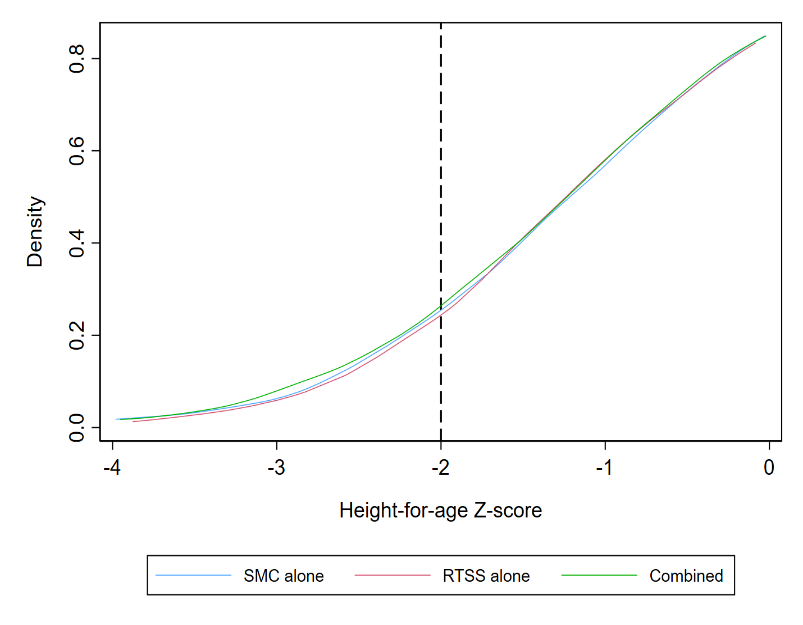

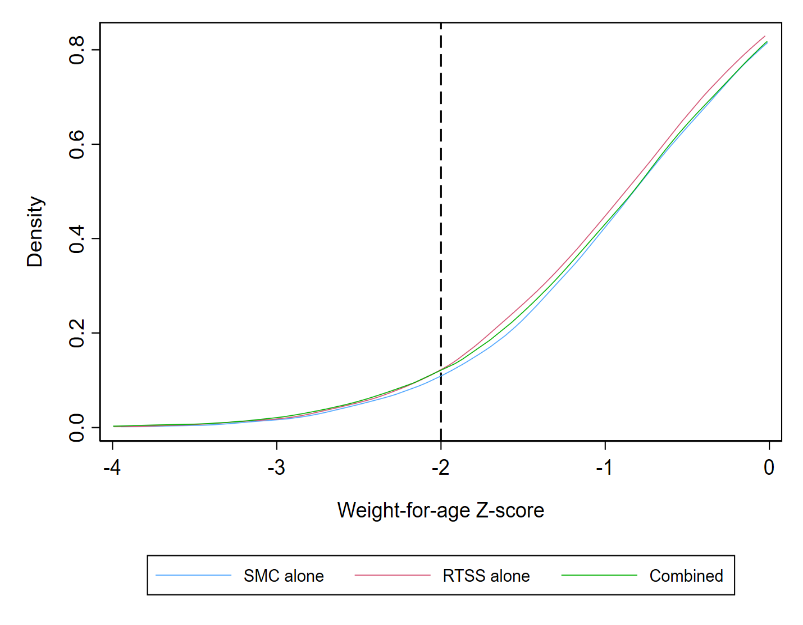

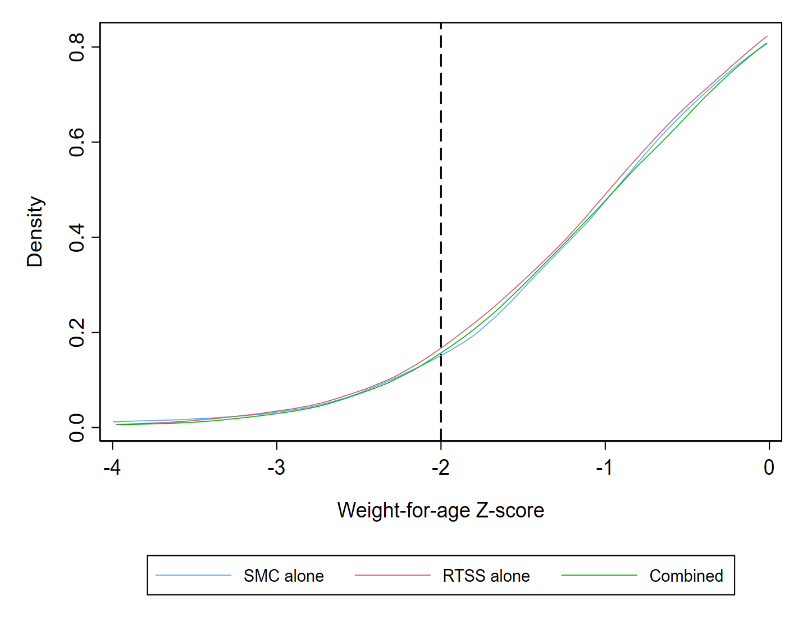

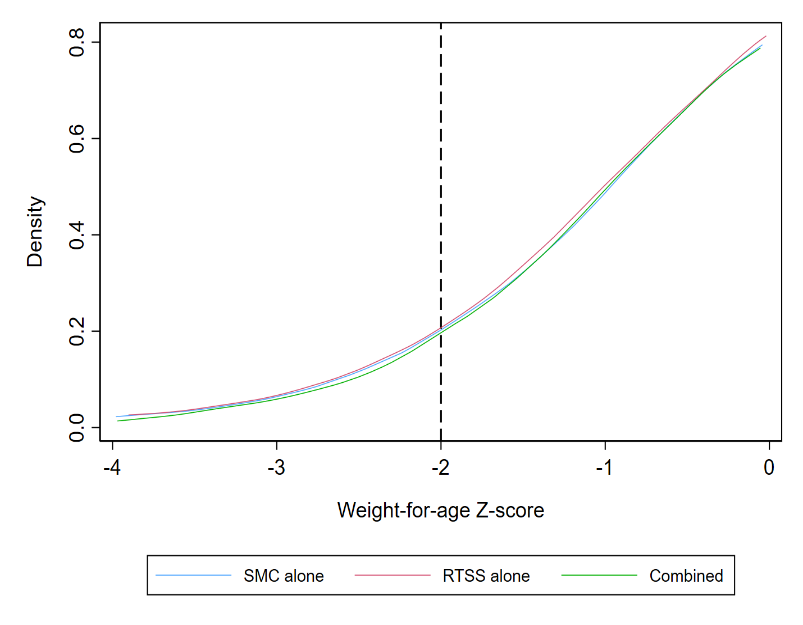

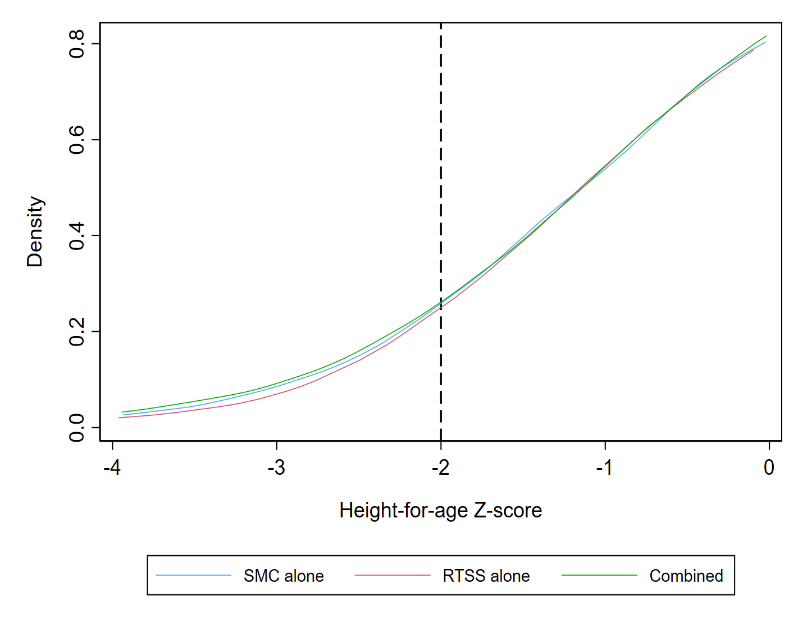

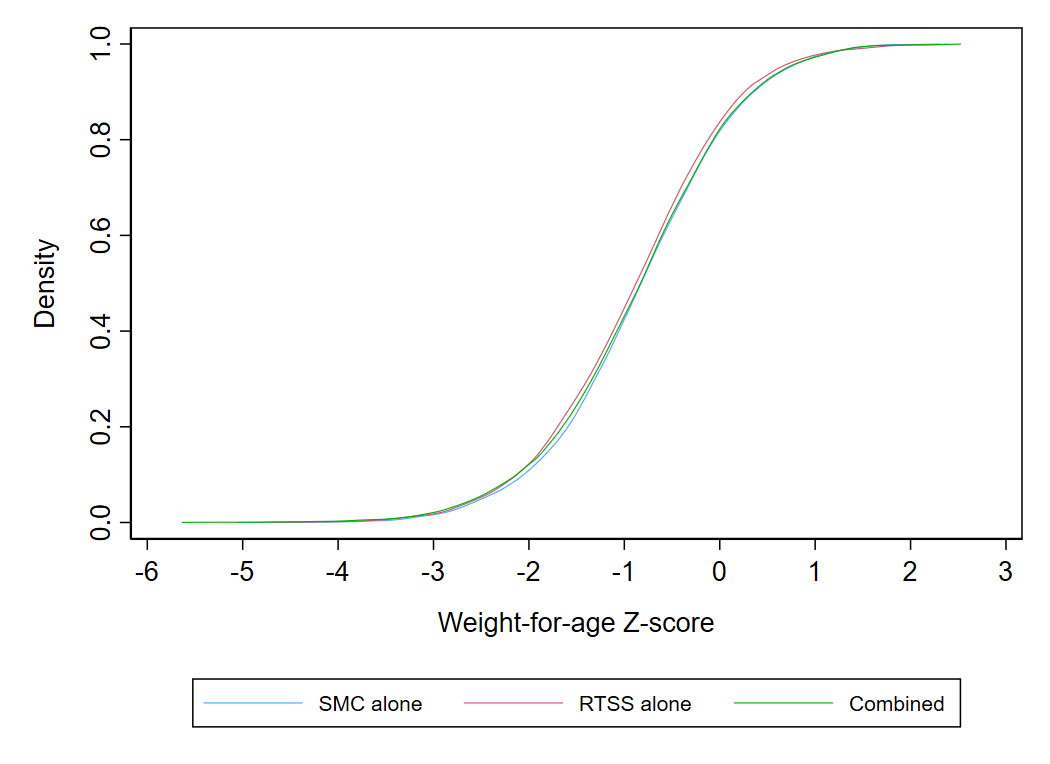


**2019**

**2018**

**Table S2. Difference in mean changes in anthropometric measurements between study arms in Burkina Faso and Mali at the end of transmission season surveys between 2017-2018, 2018-2019 and 2017-2019**

|  | **Arm** | **Mean (SD), N** | **Difference in mean change (95% CI) RTS,S alone or Combined group vs. SMC alone** | **Difference in mean change (95% CI) Combined group vs. RTS,S alone** |
| --- | --- | --- | --- | --- |
|  |  |  |  |  |
| **Change in weight (kg)** | | | | |
| 2017-2018 | SMC alone | 2.123 (1.11), 1493 | Reference |  |
|  | RTS,S alone | 2.091 (1.07), 1533 | -0.033 (-0.111, 0.044) | Reference |
|  | Combined | 2.107 (1.08), 1503 | -0.018 (-0.095, 0.060) | 0.016 (-0.061, 0.093) |
|  |  |  |  |  |
| 2018-2019 | SMC alone | 2.000 (1.10), 1486 | Reference |  |
|  | RTS,S alone | 1.963 (1.13), 1518 | -0.036 (-0.116, 0.044) | Reference |
|  | Combined | 1.944 (1.11), 1519 | -0.055 (-0.135, 0.025) | -0.019 (-0.098, 0.060) |
|  |  |  |  |  |
| 2017-2019 | SMC alone | 4.112 (1.26), 1461 | Reference |  |
|  | RTS,S alone | 4.046 (1.25), 1467 | -0.067 (-0.158, 0.023) | Reference |
|  | Combined | 4.056 (1.24), 1469 | -0.057 (-0.148, 0.033) | 0.010 (-0.080, 0.100) |
|  |  |  |  |  |
| **Change in height (cm)** | | | | |
| 2017-2018 | SMC alone | 8.488 (3.27), 1490 | Reference |  |
|  | RTS,S alone | 8.448 (3.15), 1532 | -0.048 (-0.278, 0.182) | Reference |
|  | Combined | 8.578 (3.29), 1499 | 0.081 (-0.150, 0.312) | 0.129 (-0.101, 0.358) |
|  |  |  |  |  |
| 2018-2019 | SMC alone | 8.063 (2.89), 1485 | Reference |  |
|  | RTS,S alone | 7.996 (2.81), 1518 | -0.079 (-0.283, 0.124) | Reference |
|  | Combined | 8.017 (2.87), 1518 | -0.050 (-0.254, 0.153) | 0.029 (-0.174, 0.231) |
|  |  |  |  |  |
| 2017-2019 | SMC alone | 16.458 (3.61), 1458 | Reference |  |
|  | RTS,S alone | 16.25 (3.69), 1466 | -0.035 (-0.298, 0.228) | Reference |
|  | Combined | 16.622 (3.7), 1465 | 0.136 (-0.127, 0.399) | 0.171 (-0.091, 0.434) |
|  |  |  |  |  |
| **Change in MUAC (mm)** | | | | |
| 2017-2018 | SMC alone | 4.183 (9.42), 1537 | Reference |  |
|  | RTS,S alone | 4.455 (9.24), 1589 | 0.269 (-0.402, 0.940) | Reference |
|  | Combined | 4.754 (10.04), 1553 | 0.568 (-0.107, 1.243) | 0.299 (-0.370, 0.969) |
|  |  |  |  |  |
| 2018-2019 | SMC alone | 6.492 (8.87), 1521 | Reference |  |
|  | RTS,S alone | 6.163 (9.04), 1558 | -0.356 (-1.002, 0.290) | Reference |
|  | Combined | 5.975 (9.55), 1551 | -0.526 (-1.173, 0.121) | -0.170 (-0.813, 0.473) |
|  |  |  |  |  |
| 2017-2019 | SMC alone | 10.879 (10.24), 1502 | Reference |  |
|  | RTS,S alone | 10.743 (9.98), 1512 | -0.166 (-0.905, 0.573) | Reference |
|  | Combined | 10.869 (10.84), 1508 | -0.034 (-0.774, 0.705) | 0.131 (-0.607, 0.869) |

**Table S3. Test of interaction between country and treatment arm**

|  | **P value** | | |
| --- | --- | --- | --- |
|  | **2017** | **2018** | **2019** |
| **Wasted** | 0.1481 | 0.6624 | 0.8805 |
| **Severely wasted** | 0.6102 | 0.2385 | 0.7197 |
| **Stunted** | 0.8327 | 0.4457 | 0.6813 |
| **Severely stunted** | 0.2479 | 0.4805 | 0.8788 |

**Figure S4. Prevalence of key nutritional outcomes in study children between study arms (SMC, RTS,S/AS01_E_ and combined SMC + RTS,S/AS01_E_) in Burkina Faso (a) and Mali (b) over the study period 2017-2019 (mITT population)**

1. Burkina Faso


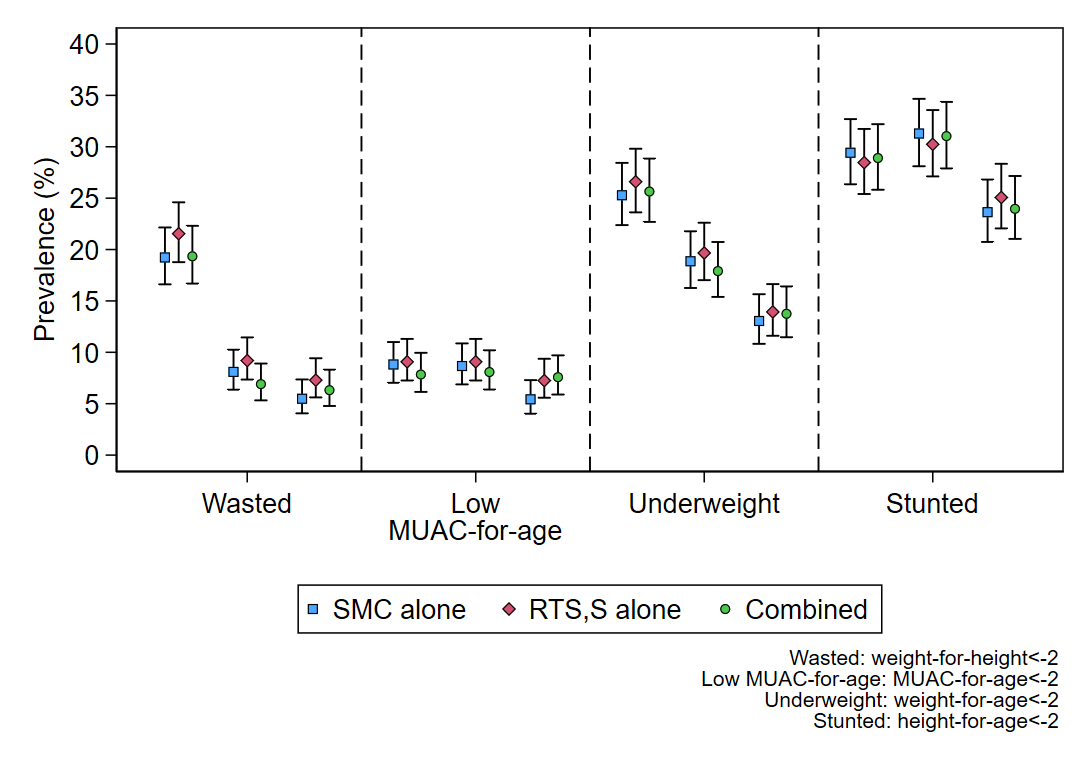

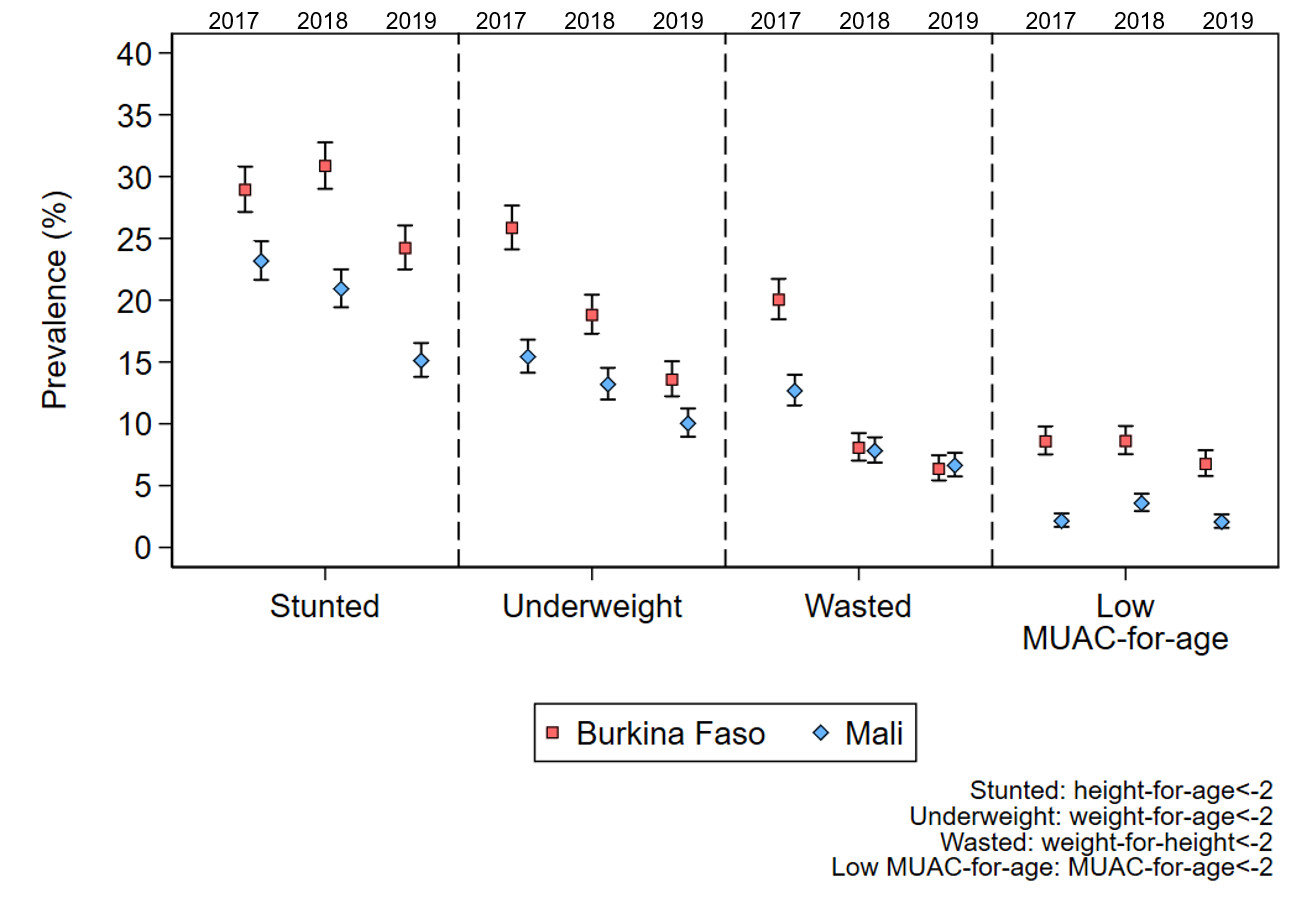

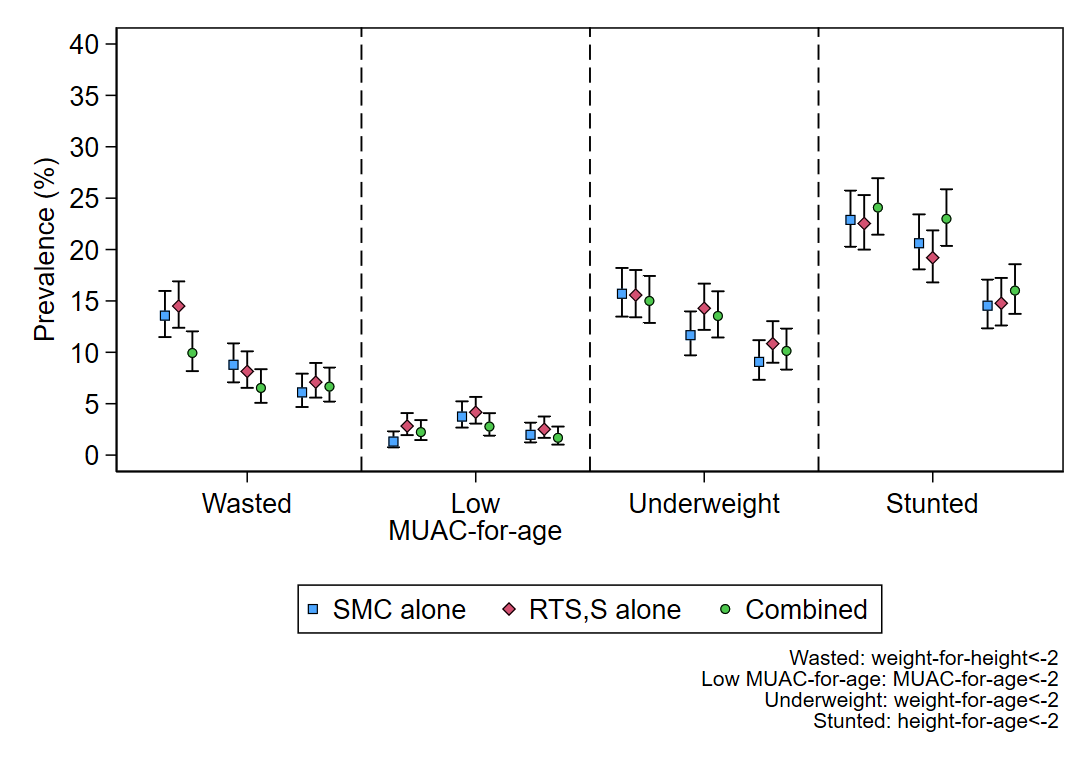

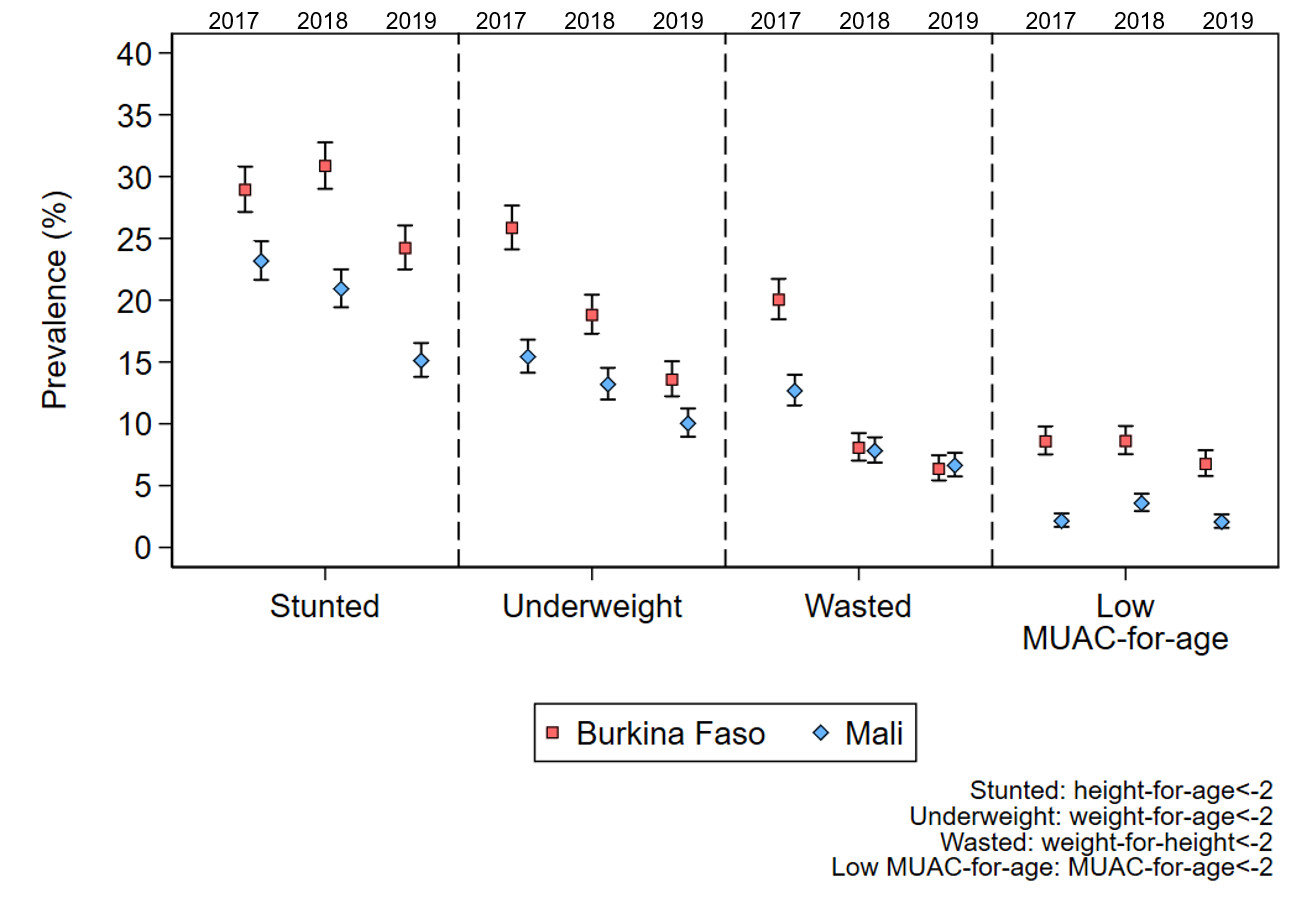


1. Mali

**Table S4. Prevalence and prevalence ratios of primary outcomes between study arms in Burkina Faso and Mali at the end of the malaria transmission season surveys (per protocol population)**

| **Group** | **n/N(%)** | **Prevalence Ratio (95% CI) RTS,S alone or Combined groups vs. SMC alone** | **Prevalence Ratio (95% CI) Combined vs. RTS,S alone** | **Global test of homogeneity** | **P value**  **vs SMC alone** | **P value vs RTS,S alone** |
| --- | --- | --- | --- | --- | --- | --- |
| **Wasted (WHZ < -2) in 2017** | | | | | | |
| SMC alone | 230/1414 (16.3) | Reference |  |  |  |  |
| RTS,S alone | 237/1425 (16.6) | 1.02 (0.87, 1.21) | Reference | 0.071 | 0.79 |  |
| Combined | 196/1422 (13.8) | 0.85 (0.71, 1.01) | 0.83 (0.70, 0.98) |  | 0.061 | 0.032 |
|  |  |  |  |  |  |  |
| **Stunted (HAZ < -2) in 2019** | | | | | | |
| SMC alone | 282/1518 (18.6) | Reference |  |  |  |  |
| RTS,S alone | 290/1535 (18.9) | 1.03 (0.89, 1.19) | Reference | 0.70 | 0.71 |  |
| Combined | 303/1536 (19.7) | 1.06 (0.92, 1.23) | 1.04 (0.90, 1.20) |  | 0.40 | 0.63 |
